# UHPLC-HRMS/MS Chemical Fingerprinting of the Bioactive Partition from Cultivated *Piper aduncum* L.

**DOI:** 10.3390/molecules29081690

**Published:** 2024-04-09

**Authors:** Adélia Viviane de Luna, Thayssa da Silva Ferreira Fagundes, Ygor Jessé Ramos, Marlon Heggdorne de Araújo, Michelle Frazão Muzitano, Sanderson Dias Calixto, Thatiana Lopes Biá Ventura Simão, George Azevedo de Queiroz, Elsie Franklin Guimarães, André Mesquita Marques, Davyson de Lima Moreira

**Affiliations:** 1 Postgraduate Program in Translational Research in Drugs and Medicines, Pharmaceutical Technology Institute, Far-Manguinhos, Fiocruz, Rua Sizenando Nabuco, 100, Manguinhos, Rio de Janeiro 21041-250, RJ, Brazil; lunaviviane31@gmail.com (A.V.d.L.); andrefarmaciarj@yahoo.com.br (A.M.M.); 2 Botanical Garden Research Institute of Rio de Janeiro, Rua Pacheco Leão, 915, Jardim Botânico, Rio de Janeiro 22460-030, RJ, Brazil; thayssasffagundes@gmail.com (T.d.S.F.F.); georgeazevedo08@gmail.com (G.A.d.Q.); eguimar@jbrj.gov.br (E.F.G.); 3 Marine Biotechnology Departament, Almirante Paulo Moreira Institute of Marine Studies, Rua Kioto, 253, Arraial do Cabo, Rio de Janeiro 28930-000, RJ, Brazil; 4 Farmácia da Terra Laboratory, Faculty of Pharmacy, Federal University of Bahia, Rua Barão de Jeremoabo, 147, Ondina, Salvador 40170-115, BA, Brazil; 5 Laboratory of Bioatives Products, Institute of Pharmaceutical Sciences, Federal University of Rio de Janeiro, Rua Alcides da Conceição, 159, Macaé 27933-378, RJ, Brazil; marlon.heggdorne@gmail.com (M.H.d.A.); mfmuzitano@macae.ufrj.br (M.F.M.); 6 Recenor Biology Laboratory, Center of Biosciences and Biotechnology, State University of North Fluminense Darcy Ribeiro, Rua Alberto Lamego, 2000, Campos dos Goytacazes 28013-602, RJ, Brazil; sandersoncalixto@yahoo.com.br (S.D.C.); thativentura@uenf.com (T.L.B.V.S.); 7 Pharmacy Departament, State University of Rio de janeiro, Manuel Caldeira de Alvarenga 1203 st, Campo Grande, Rio de Janeiro 23070-200, RJ, Brazil

**Keywords:** Piperaceae, antimycobacterial, *Mycobacterium tuberculosis*, GNPS, molecular network, agroecological cultivation, medicinal plant

## Abstract

*Piper aduncum* L. is widely distributed in tropical regions and the ethnobotanical uses of this species encompass medicinal applications for the treatment of respiratory, antimicrobial, and gynecological diseases. Chemical studies reveal a diverse array of secondary metabolites, including terpenes, flavonoids, and prenylated compounds. Extracts from *P. aduncum* have shown antibacterial, antifungal, and larvicidal activities. Our study explores the activity of extracts and partitions against *Mycobacterium tuberculosis* H37Rv, as well as the chemical diversity of the bioactive partition. This marks the first investigation of the bioactive partition of *P. aduncum* from agroecological cultivation. The ethyl acetate partition from the ethanolic leaf extract (PAEPL) was found to be the most active. PAEPL was subjected to column chromatography using Sephadex LH-20 and the obtained fractions were analyzed using UHPLC-HRMS/MS. The MS/MS data from the fractions were submitted to the online GNPS platform for the generation of the molecular network, which displayed 1714 nodes and 167 clusters. Compounds were identified via manual inspection and different libraries, allowing the annotation of 83 compounds, including flavonoids, benzoic acid derivatives, glycosides, free fatty acids, and glycerol-esterified fatty acids. This study provides the first chemical fingerprint of an antimycobacterial sample from *P. aduncum* cultivated in an agroecological system.

## 1. Introduction

*Piper aduncum* L., belonging to the Piperaceae family, is a species widely distributed in tropical regions, with a relevant presence in the Americas [1,2]. This plant is characterized by its leaves with secondary veins reaching the middle portion of the blade and a curved inflorescence, distinguishing it from *P. mollicomum* by its pubescent leaves and trichomes that feel rough to the touch. These morphological characteristics not only aid in its botanical identification but also suggest an ecological adaptation to the diverse environmental conditions found in its extensive habitat [3].

This *Piper* species shows a diversified ethnobotanical use, encompassing medicinal applications and traditional practices in many countries in the Pacific, Latin American, and Caribbean regions. Historically, its leaves have been used as an astringent, digestive stimulant, diuretic, antimalarial, sedative, and laxative agent [4,5,6]. In Brazil, it is widely cultivated for the extraction of its essential oil and employed as an antimicrobial and antiparasitic agent [7,8,9].

Chemical studies have shown a rich diversity of secondary metabolites in the wild *P. aduncum* samples. These include terpenoids, alkaloids, amides, flavonoids (chalcones, flavones, and flavanones), cinnamic acid derivatives, benzoic acid derivatives, chromenes, and prenylated compounds [5,6,7,10,11,12,13,14]. However, there have been no phytochemical investigations of the non-volatile constituents with cultivated specimens of *P. aduncum*.

The biological and pharmacological activities associated with *P. aduncum* extracts are extensive and noteworthy. Pharmacological studies have demonstrated the effectiveness of these extracts in antibacterial, antifungal, antiprotozoal, larvicidal, insecticidal, molluscicidal, cytotoxic, antidepressant, and anxiolytic activities [4,5,6,9,15]. The wide range of biological activities described highlights the multifunctionality of this species and warrants exploration to isolate specific bioactive small molecules. This potential becomes even more evident considering that this species undergoes standardized cultivation in agroecological systems [16]. In this way, exploring new compounds in *Piper* species may lead to the discovery of novel compounds, particularly those with antimicrobial properties.

The urgency to discover new antimicrobial agents is underscored by the global challenge posed by *Mycobacterium tuberculosis* H37Rv, a strain of high virulence. Tuberculosis, caused by *M. tuberculosis*, remains a major public health concern, with recent WHO data reporting approximately 10.6 million infections and 1.3 million deaths in 2022 alone [16,17. The disease is the second leading cause of death from infectious diseases, with the issue of multi-drug resistance exacerbating the public health crisis [17].

In this study, we investigate the chemical diversity present in the ethyl acetate partition from the ethanolic extract, which has demonstrated activity against *Mycobacterium tuberculosis* H37Rv. It is worth noting that this marks the first examination of the chemical composition of a bioactive partition from *P. aduncum* against *M. tuberculosis*, with leaf extract obtained from cultivation standardized by the group in an agroecological environment [16].

## 2. Results and Discussion

### 2.1. Activity against Mycobacterium tuberculosis H37Rv

**Figure 1:**
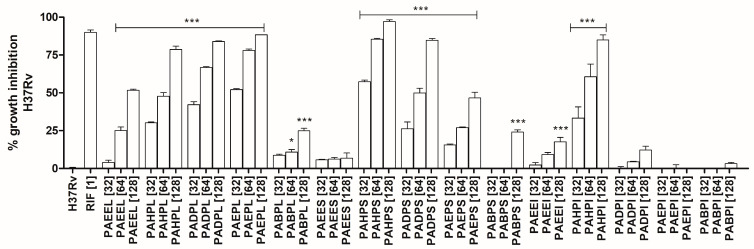
Inhibition of *Mycobacterium tuberculosis* H37Rv growth after treatment with extracts and partitions of *Piper aduncum*. MTT assay after 5 days of incubation in the presence of samples at concentrations of 32, 64, and 128 µg/mL. Positive control: *M. tuberculosis* H37Rv (1 × 10^6^ CFU/mL) treated with rifampicin (treatment drug), and negative control *M. tuberculosis* H37Rv untreated (1 × 10^6^ CFU/mL). Statistical analysis: One-way ANOVA followed by the Tukey test. *** *p* < 0.001 and * *p* < 0.05 compared to negative control. Triplicate results are represented as mean ± standard error. RIF—Rifampicin; PAEEL—Ethanolic extract from leaves; PAHPL—Hexane partition from leaves’ ethanolic extract; PADPL—Dichloromethane partition from leaves’ ethanolic extract; PAEPL—Ethyl acetate partition from leaves’ ethanolic extract; PABPL—Butanol partition from leaves’ ethanolic extract; PAEES—Ethanolic extract from stems; PAHPS—Hexane partition from stems’ ethanolic extract; PADPS—Dichloromethane partition from stems’ ethanolic extract; PAEPS—Ethyl acetate partition from stems’ ethanolic extract; PABPS—Butanol partition from stems’ ethanolic extract; PAEEI—Ethanolic extract from inflorescences; PAHPI—Hexane partition from inflorescences’ ethanolic extract; PADPI—Dichloromethane partition from inflorescences’ ethanolic extract; PAEPI—Ethyl acetate partition from inflorescences’ ethanolic extract; PABPI—Butanol partition from inflorescences’ ethanolic extract.

The results of the growth inhibition tests against *M. tuberculosis* H37Rv for extracts and partitions of *P. aduncum* are shown in Table 1 and Figure 1.

According to Table 1 and Figure 1, the most active samples were the hexane partition from the ethanolic stem extract (PAHPS, 29.74 ± 1.01) and the ethyl acetate partition from the ethanolic leaf extract (PAEPL, 27.98 ± 1.01). Due to the relevant antimicrobial activity and a larger amount of material, PAEPL was purified using Sephadex-LH20 column chromatography, yielding five fractions referred to as SFR1—SFR5. SFR1 did not show the presence of any compounds in the analysis by TLC. Therefore, SFR2—SFR5 were subjected to analysis by UHPLC-HRMS/MS, as described in the experimental section.

### 2.2. Chemical Composition Analysis of the Bioactive Partition

**Figure 2:**
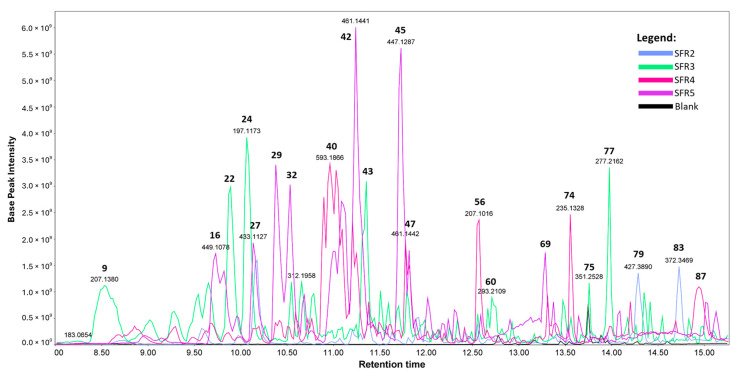
Overlaid chromatograms of base peak recorded (Rt 8.0 to 16.5 min) with UHPLC-HRMS/MS in positive ionization mode of fractions 2 to 5 (SFR2-SFR5) obtained using Sephadex LH-20 column chromatography of the bioactive ethyl acetate partition from the ethanolic extract of *Piper aduncum* L. The bold numbers in the peaks shown correspond to the substances described in the text and Table 2.

The UHPLC-HRMS/MS analyses in positive ionization mode afforded more comprehensive information, prompting their selection for ion investigation. The overlaid chromatograms of all fractions from the bioactive ethyl acetate partition against *M. tuberculosis* are depicted in Figure 2. Fraction 1, eluted from the Sephadex LH-20 chromatographic column, was excluded from this study as it did not contain any compound.

The MS^2^ data from the fractions of the ethyl acetate partition (PAEPL) acquired in positive mode were submitted to the online GNPS platform for the generation of the molecular network. After removing ions present in the blank (mobile phase), the resulting molecular network displayed a total of **1714 nodes** and **167 clusters** formed by at least two ions with similarity in the MS, using a cosine score of 0.75.

The data analysis, aided by manual inspection and/ or different libraries, as well as the specialized literature about the chemistry of the Piperaceae family, led to the annotation of **83 compounds** (error up to ± 5 ppm), with 54 identified based on library suggestions and 29 after manual inspection of the data, all tentatively confirmed through fragmentation profiles. Among the annotated compound classes, non-glycosylated and glycosylated flavonoids, chromenes, cinnamic acid derivatives, amides, glycosides, glycerides, and benzoic acid derivatives (including prenylated) stand out.

**Figure 3:**
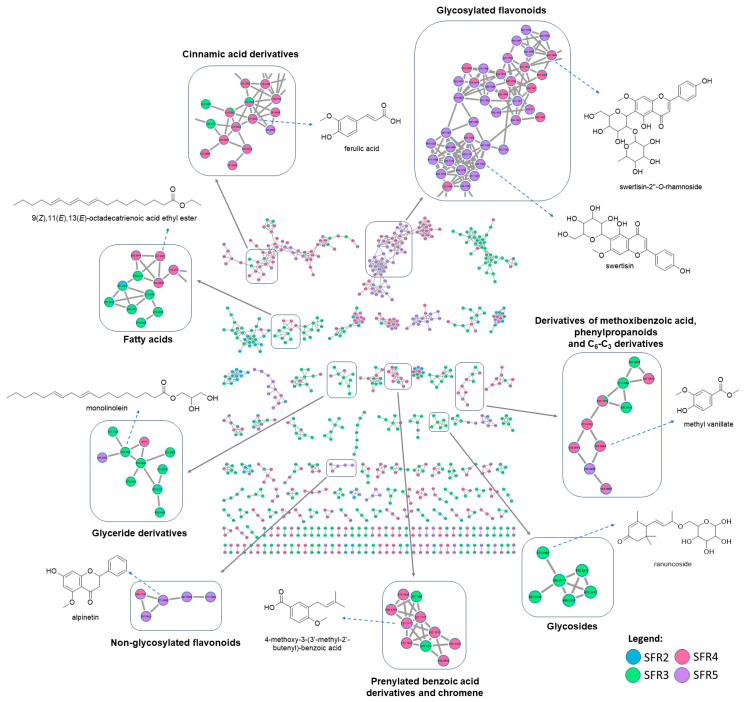
Molecular network of fractions 2 to 5 (SFR2–SFR5) obtained by Sephadex LH-20 column chromatography of the bioactive ethyl acetate partition from the ethanolic extract of *Piper aduncum* L. Only clusters containing at least two nodes are shown. Nodes present in the blank (mobile phase) were excluded. The eight annotated molecular families are highlighted, with the chemical structure of at least one example from each family.

Figure 3 shows the molecular network generated from GNPS, highlighting the eight annotated molecular families according to their chemical classes. The annotated substances are listed in Table 2.

The family of glycosylated flavonoids (Figure 3 and Supplementary Figures S1 and S2) presented 71 nodes where 12 monoglycosyl flavonoids (**16**, **23**, **27**, **29**, **30**, **35**, **38**, **41**, **45**, **47**, **51**, and **53**) and **10 diglycosyl ones** (**17**, **20**, **21**, **25**, **26**, **28**, **31**, **37**, **39**, and **40**) were annotated. Swertisin (**45**, *m*/*z* 447.1287, [M+H]^+^) was recorded as one of the major monoglycosylated flavonoids in SFR5, while swertisin-2″-*O*-rhamnoside (**40**, *m*/*z* 593.1866, [M+H]^+^) was predominant in SFR4. Monoglycosylated flavonoids, including **40** and **45**, were characterized by the presence of fragment ions [M+H-150]^+^ and [M+H-120]^+^, representing the loss of the glycosyl moiety in flavonoids via the retro-Diels–Alder fragmentation mechanism [18]. For diglycosylated flavonoids, the main fragments recorded in the fragmentation spectrum correspond to the sequential loss of glycosyl moieties, i.e., ([M+H]^+^)-glycosyl-1 → ([M+H]^+^)-glycosyl-2. It is noteworthy that glycosylated flavonoids are common in species of the genus *Piper*, including in *P. aduncum* [19,20,21,22].

Another molecular family was annotated, exclusively consisting of non-glycosylated flavonoids (Figure 3 and Figure S3), composed of five nodes identified as alpinetin (**54**, *m*/*z* 271.0959, [M+H]^+^), eriodictyol-7,3′-dimethyl ether (**62**, *m*/*z* 317.1009, [M+H]^+^), sakuranetin (**63**, *m*/*z* 287.0909, [M+H]^+^), pinocembrine (**65**, *m*/*z* 257.0831, [M+H]^+^), and 5,7-dimethoxyflavanone (**66**, *m*/*z* 285.1134, [M+H]^+^). These flavonoids were recorded mainly in the SFR5 fraction, except for 5,7-dimethoxyflavanone (**66**), which was recorded in higher percentage contents in the SFR4 fraction. These non-glycosylated flavonoids were characterized by the formation of a fragment resulting from the breaking of the C ring via retro-Diels–Alder fragmentation, considered the most important mechanism for annotating this class of compounds [18,23]. Additionally, the MS^2^ fragmentation spectrum of these substances showed the base ion (100%) as the [M+H]^+^ adduct. The five annotated flavonoids have been previously identified in species of the *Piper* genus [24,25].

The molecular family of cinnamic acid derivatives (Figure 3 and Figure S4) presented 71 nodes where eight **precursor ions represented** by substances **11**, **14**, **18**, **19**, **46**, **48**, **49**, and **52** were annotated, with higher occurrences in SFR4. The compounds in this molecular family were characterized by the loss of some neutral molecules, such as H_2_O, CH_3_OH, and C=O. Ferulic acid was annotated as the precursor ion [M+H]^+^ (**19**, *m*/*z* 195.0653) and also in the form of the adduct [M + H − H_2_O]+ (**18**, *m*/*z* 177.0546). In both cases, the MS^2^ fragmentation profile was the same, mainly presenting product ions at *m*/*z* 177 ([M + H—H_2_O]^+^), *m*/*z* 149 ([M + H − H_2_O − C=O]+), *m*/*z* 145 ([M + H − H_2_O − CH_3_OH]+), and *m*/*z* 117 ([M + H − H_2_O − C=O − CH_3_OH]+), as described by [26]. Ferulic acid, like other cinnamic acid derivatives, is common in species of the *Piper* genus [27,28].

This GNPS analysis generated a family represented by prenylated derivatives of benzoic acid (Figure 3 and Figure S5), consisting of 10 nodes where six **substances** (**55**, **56**, **68**, **70**, **73**, and **74**) were annotated, including a chromene, 2,2-dimethyl-8-(3-methylbut-2-*en*-1-yl)-2H-chromene-6-carboxylic acid (**68**, *m*/*z* 273.1497, [M+H]^+^). The 4-hydroxy-3-(3′-methyl-2′-butenyl)-benzoic acid (**56**, *m*/*z* 207.1012, [M+H]^+^) is one of the main constituents in SFR4. The main fragment registered in the MS^2^ spectra of this molecular family indicates the loss of the neutral fragment consisting of a prenyl group with 56 mass units. The class of the prenylated derivatives of benzoic acid is known in the species *P. aduncum* [13,29], including the annotated chromene **68** [10].

In the molecular family mostly composed of methoxybenzoic acid derivatives, as well as phenylpropanoids and C_6_-C_3_ derivatives, consisting of 10 nodes (Figure 3 and Figure S6), nine **compounds** (**1**, **3**, **5**, **7**, **8**, **10**, **15**, **36**, and **44**) were annotated in the SFR3, SFR4, and SFR5 fractions. The MS^2^ spectra of these substances showed two main ions: (a) one formed from radical fragmentation and (b) another that characterizes the neutral elimination of a methanol molecule ([M+H-CH_3_OH]^+^), which is generated from the fragmentation of the ester group [18]. For instance, methyl vanillate (**3**, *m*/*z* 183.0664, [M+H]^+^) was annotated using the GNPS library and presented the main fragments *m*/*z* 151 ([M + H − CH_3_OH]^+^) and *m*/*z* 124 (C_7_H_8_O_2_^+^). Meanwhile, 6-methoxy eugenol (**44**, *m*/*z* 195.1016, [M+H]^+^), a phenylpropanoid, only showed the radical fragment *m*/*z* 154 (C_8_H_10_O_3_^+^) as the major ion in the MS^2^ spectrum. These compounds are common in Piperaceae species [20], and substance **44** has already been described in *Piper* species [30]. However, this is the first description of methyl vanillate (3) in this genus.

In another molecular family composed of six nodes, three **glycosides** were annotated, namely, dihydroroseoside (**12**, *m*/*z* 386.2171, [M+H]^+^), roseoside (**13**, *m*/*z* 371.2065, [M+H]^+^), and ranuncoside (**34**, *m*/*z* 387.2016, [M+H]^+^), found in the SFR3 fraction (Figure 3 and Figure S7). Dihydroroseoside (**12**), for instance, was annotated using the GNPS library and exhibited the main fragment *m*/*z* 209 (C_13_H_21_O_2_^+^), resulting from the loss of glucose. Substances **12** and **13** have been previously described for the *Piper* genus [31,32]; however, this is the first description of ranuncoside (**34**) for the genus.

The molecular cluster of glycerides (Figure 3 and Figure S8) presented 10 nodes found in SFR3. In this family, monoolein (**80**, *m*/*z* 357.3000, [M+H]^+^), monolinolenin (**86**, *m*/*z* 353.2683, [M+H]^+^), monolinolein (**88**, *m*/*z* 355.2840, [M+H]^+^), 2,3-dihydroxypropyl-6,9,12,15-octadecatetraenoate (**75**, *m*/*z* 351.2528, [M+H]^+^), and 9,12,13-trihydroxyoctadeca-10,15-dienoic acid (**61**, *m*/*z* 311.2216, [M+H]^+^) were identified. Fatty acid derivatives were characterized by the presence of fragment ions indicating the subsequent loss of CH_2_ units, as well as the ion [M+H-92]^+^ corresponding to the elimination of the triol group [33,34]. So far, there is no description of these compounds in the *Piper* genus.

In another molecular family consisting of 21 nodes, the GNPS library annotated seven **substances**, including five fatty acids (**59**, **71**, **77**, **78**, and **82**) and two fatty acid esters (**84, 89**) (Figure 3 and Figure S9). The substance 9-hydroxy-10,12,15-octadecatrienoic acid (**77**, *m*/*z* 277.2160, [M + H − H_2_O]^+^) is the second most abundant constituent in SFR3, and there are no previous reports of the occurrence of this compound in *Piper* species. These substances are known as linoleic acids and their derivatives. For example, a methyl ester of **77** isolated from the leaves of *Ehretia dicksonii* Hance (Boraginaceae) demonstrated interesting in vivo anti-inflammatory activity [35].

Other compounds of various classes (Supplementary Figure S10) were annotated based on the GNPS library, forming clusters of two or three nodes, or in the form of self-loops (without any spectral similarity with other ions):

(a) The monoterpenic lactone loliolide (**24**, *m*/*z* 197.1174, [M+H]^+^), a major constituent of SFR3, with seven fragment ions matching the GNPS library spectrum, previously described in the species *Piper boehmeriifolium* (Miq.) Wall. ex C.DC. [36];
(b) The flavonoids wogonin (**69**, *m*/*z* 285.0758, [M+H]^+^) in SFR5, along with two glycosylated flavonoids, isoswertisin (**32**, *m*/*z* 447.1281, [M+H]^+^) and 7-(β-D-glucopyranosyloxy)-5-methoxy-2-(4-methoxyphenyl)-4H-1-benzopyran-4-one (**42**, *m*/*z* 461.1443, [M+H]^+^), with the latter being predominant in SFR5. These flavonoids did not cluster into the corresponding molecular families due to the low-intensity signals in the MS^2^ spectrum, preventing the calculation of similarity between spectra. The presence of fragment ions was observed in the raw data, allowing for the manual annotation of substances based on the similarity with MS^2^ spectra from libraries;
(c) Two chalcones, 2,6-dihydroxy-4-methoxydihydrochalcone (**57**, *m*/*z* 273.1124, [M+H]^+^) and 2,4-dihydroxy-6-methoxydihydrochalcone (**72**, *m*/*z* 273.1118, [M+H]^+^), were identified in the SFR4 and SFR5 fractions, both with eight ions matching the spectra in the GNPS library. The analog of **57**, 2,6-dihydroxy-4-methoxychalcone was previously isolated from *P. aduncum* by our group and exhibited great leishmanicidal activity [37,38];
(d) Esculetin (**2**, *m*/*z* 179.0343, [M+H]^+^), a coumarin, was identified in SFR5, consistent with five fragment ions from the GNPS library. This coumarin is common in the plant kingdom, for example in *Artemisia capillaris* Herba (Asteraceae), which has shown interesting anticonvulsant activity in vivo [39]. However, it is the first description of esculetin (**2**) in *Piper*. Indeed, coumarins are not typically associated with the *Piper* genus. Nonetheless, some articles have suggested the presence of this class in *Piper* [40];
(e) Three amides, 9-octadecenamide (**76**, *m*/*z* 282.2791, [M+H]^+^), 13-docosenamide (**81**, *m*/*z* 338.3416, [M+H]^+^), both with twelve fragment ions consistent with the GNPS library, and pipzorine (**85**, *m*/*z* 364.3574, [M+H]^+^), are present in SFR3, SFR4, and SFR5. The presence of pipzorine in SFR5 was inferred through manual annotation propagation, based on the spectra of the other two amides present in the same molecular cluster. Amides, including pipzorine, are commonly found in the *Piper* genus [20,41]. However, this is the first description of amides **76** and **81** for this genus;
(f) The piperamides piperlonguminine (**64**, *m*/*z* 274.1453, [M+H]^+^) and piperine (**67**, *m*/*z* 286.1436, [M+H]^+^) were detected in SFR4 and SFR5, with eight and twelve fragment ions, respectively, consistent with the GNPS library. Piperlonguminine (**64**) has shown interesting in vivo antitumor activity [42], and piperine (**67**) exhibits various pharmacological effects, including antiproliferative, antitumor, antiangiogenic, antioxidant, antidiabetic, anti-obesity, cardioprotective, antimicrobial, anti-aging, and immunomodulatory properties in various in vitro and in vivo experimental assays [43]. Additionally, compound **67** has demonstrated antiparasitic, hepatoprotective, antiallergic, anti-inflammatory, and neuroprotective properties [44]. Piperamides are common in *Piper* [20,45]; however, this is the first description of these compounds in *P. aduncum*;
(g) Other phenolic compounds such as vanillic acid (**4**, *m*/*z* 169.0498, [M+H]^+^), vanillin (**6**, *m*/*z* 153.0547, [M+H]^+^), sinapaldehyde (**33**, *m*/*z* 209.0808, [M+H]^+^), and ethyl vanillate (**50**, *m*/*z* 197.0811, [M+H]^+^) were detected in SFR4 and SFR5, all with six fragment ions corresponding to the GNPS library. Compounds **4** and **6** are widespread in the plant kingdom. Vanillic acid (**4**) is well-known for its pharmacological properties such as antioxidant, anti-inflammatory, immunostimulant, neuroprotective, hepatoprotective, cardioprotective, and antiapoptotic effects. It has also been reported to have the potential to attenuate Aβ1-42-induced cognitive impairment and oxidative stress, contributing to the treatment of Alzheimer’s disease [44]. Vanillin (**6**) also exhibits anticancer, antidiabetic, anti-inflammatory, and antimicrobial activities [46].

It is quite challenging to correlate the activity against *M. tuberculosis* with a chemically complex partition. However, some inferences can be made. For instance, the ethyl acetate partition of the ethanolic extract from the leaves of *P. aduncum* proved to be rich in flavonoids, with 30 substances belonging to this class of phenolics being annotated. According to [47], flavonoids have significant inhibitory potential against mycobacterial activity, acting on the inhibition of the proteasome and the inhibition of nitric oxide formation.

Considering the chromatogram in Figure 2, the major compounds identified in the fractions were orientin (**16**), loliolide (**24**), vitexin (**27**), isovitexin (**29**), isoswertisin (**32**), swertisin-2″-*O*-rhamnoside (**40**), 7-(β-D-glucopyranosyloxy)-5-methoxy-2-(4-methoxyphenyl)-4H-1-benzopyran-4-one (**42**), swertisin (**45**), embigenin (**47**), 4-hydroxy-3-(3′-methyl-2′-butenyl)-benzoic acid (**56**), wogonin (**69**), methyl-4-methoxy-3-(3′-methyl-2′-butenyl) benzoate (**74**), and 9-hydroxy-10,12,15-octadecatrienoic acid (**77**). Among these, the flavonoids orientin (**16**) and vitexin (**27**) stand out, showing antimycobacterial activity against *M. tuberculosis* H37Rv strains, with MIC values of 160 μg/mL and 80 μg/mL, respectively [48]. The author also suggests that the *C*-glycosylation at position 8 in orientin is crucial for its action against mycobacteria. Studies described wogonin (**69**) activity against *M. smegmatis* (MIC_99_ = 128.0 mg/mL) and *M. aurum* (MIC_99_ = 31.25 mg/mL) [49]. Additionally, [50] reported a 53.97% inhibition rate against *M. tuberculosis* for wogonin (**69**). The monoterpenic lactone loliolide (**24**) also exhibited activity against *M. tuberculosis* H37Rv with a MIC_99_ value of 250.0 mg/L [51].

All the examples described here demonstrated a MIC higher than that of the ethyl acetate partition (PAEPL). This could be attributed to a synergistic effect among the compounds present in this partition.

Several biological studies have documented the antimycobacterial efficacy of essential oils derived from different species of the *Piper* genus, showcasing moderate to good activity against *M. tuberculosis*. Specifically, essential oils from the infructescences and inflorescences of *Piper lhotzkyanum* Kunth exhibited minimum inhibitory concentrations (MICs) of 76 µg/mL and 128 µg/mL, respectively [52]. Similarly, leaf oils from *Piper cernuum* Vell., *Piper diospyrifolium* Kunth, and *Piper rivinoides* Kunth demonstrated MIC values of 125 µg/mL, while *Piper mosenii* C.DC. reported an MIC of 250 µg/mL [53]. Further research indicated that oils from the roots and infructescences of *Piper multinodum* C.DC. showed MICs of 78.51 µg/mL and 85.91 µg/mL, respectively [54].

The antimicrobial potential extends beyond essential oils to extracts, fractions, and isolated compounds. The methanolic extract of *Piper guineense* Schumach. and Thonn. seeds exhibited an MIC of 256 µg/mL [55], while the ethyl acetate fraction from the methanolic extract of *Piper sarmentosum* Robx. leaves showed an MIC of 3.12 µg/mL [56]. Notably, Rukachaisirikul et al. [57,58] isolated pellitorine from hexane and methanolic extracts of *Piper sarmentosum* Roxb. fruits, demonstrating an MIC of 25 µg/mL. Similarly, the monoterpene ester (+)-borneol piperate, isolated from *Piper pedicellatum* C.DC. root extracts, exhibited an MIC of 25 µg/mL, while chabamide, isolated from *Piper chaba* Blume Hunter stem hexane extract, showed an MIC of 12.5 µg/mL [59].

Further investigations revealed the ethyl acetate fraction of the methanolic extract from *Piper taiwanense* Lin and Lu roots to possess antimycobacterial activity with an MIC of 30 µg/mL, with 4-(prop-2-enyl)1-catechol isolated from this fraction showing an MIC of 27.6 µg/mL [60]. Piperolactam and 2-oxo-16-(3′,4′-methylenedioxyphenyl) hexadecane, identified in extracts from the leaves and stems of *Piper auritum* Kunth, inhibited *M. tuberculosis* growth with MICs of 8 µg/mL and 6.25 µg/mL, respectively [61]. Scodro et al. [62] isolated three neolignans from *Piper regnellii* (Miq.) C.DC. leaf extract, with eupomatenoide-5 being the most active against *M. tuberculosis* H37Rv, and presenting an MIC of 1.9 µg/mL, thereby suggesting its potential as a candidate for future anti-TB pharmacotherapy.

Moreover, supercritical fluid extracts of *Piper diospyrifolium* (Kunth) Kunth ex Steud. leaves and a novel benzoic acid derivative were tested against the *M. tuberculosis* H37Rv strain and eight clinical isolates, showing MICs of 125 µg/mL for the H37Rv strain and ≥250 µg/mL for the clinical isolates, indicating moderate activity for this species [63]. Additionally, crude extracts and alkaloid fractions from *Piper corcovadensis* (Miq.) C.DC. roots, including isobutylamide (piperovatine), exhibited MICs of 15.6; 7.8, and 7.8 µg/mL, respectively, against the *M. tuberculosis* H37Rv strain, with MICs ranging from 0.98 to 3.9 µg/mL against clinical isolates, suggesting synergistic effects when combined with rifampicin [64]. In 2018, the antimycobacterial activity of piperine, an alkaloid found in *Piper nigrum* L. and *Piper longum* L., was evaluated, showing MICs ranging from 31.2 to 125 µg/mL. Notably, when combined with antibiotics such as rifampicin, isoniazid, ethambutol, and streptomycin, MIC values were reduced to 0.12 to 1 µg/mL, indicating a synergistic effect against evaluated clinical isolates [65].

These findings underscore the promising antimycobacterial activity of *Piper* species, though further in vivo studies and explorations of their mechanisms of action are warranted. The active compounds identified within these species are likely responsible for the observed activity, making them promising candidates for the development of new anti-TB drugs.

**Table 2:** UHPLC-HRMS-MS analysis for the chemical composition of the bioactive partition of *Piper aduncum* L.

Compound No.	Rt (min)	Precursor Ion (*m*/*z*)	Fragment Ions (MS^2^) *	Molecular Formula	Adduct Ion	Exact Mass (*m*/*z*)	Annotated Compound	Error (ppm)	Shared Peaks	Annotation Type
**1**	3.93	153.0551	153; 135; **121**; 111; 109; 107; 94; 81	C_8_H_8_O_3_	[M+H]^+^	153.05516	methyl 3-hydroxybenzoate	−0.4	-	Manual inspection
**2**	4.74	179.0343	**179**; 151; 147; 135; 133; 123	C_9_H_6_O_4_	[M+H]^+^	179.03443	esculetin	−0.7	5	GNPS library
**3**	5.37	183.0664	183; **151**; 139; 124; 107; 95; 79	C_9_H_10_O_4_	[M+H]^+^	183.06573	ethyl vanillate	3.7	5	GNPS library
**4**	5.40	169.0498	169; 151; 125; **111**; 93; 65	C_8_H_8_O_4_	[M+H]^+^	169.05008	vanillic acid	−1.7	6	GNPS library
**5**	6.80	213.0762	213; **181**; 169; 154; 149; 137; 109; 91; 81	C_10_H_12_O_5_	[M+H]^+^	213.07629	methyl 4-hydroxy-3,5-dimethoxybenzoate	−0.4	13	GNPS library
**6**	7.31	153.0547	153; 125; **111**; 93; 65	C_8_H_8_O_3_	[M+H]^+^	153.05516	vanillin	−3.0	6	GNPS library
**7**	7.65	197.0810	197; 179; 169; 165; **156**; 147; 137; 119; 97; 95; 69	C_10_H_10_O_4_	[M+H]^+^	197.08138	1-propanone, 1-(3,5-dihydroxy-4-methoxyphenyl)	−1.9	-	Manual inspection
**8**	7.82	197.0814	197; 169; 165; **156**; 147; 137; 119; 97; 69	C_10_H_12_O_4_	[M+H]^+^	197.08138	1-propanone, 1-(3,4-dihydroxy-5-methoxyphenyl)	0.1	-	Manual inspection
**9**	8.71	207.1380	207; 189; 161; 149; 123; **95**	C_13_H_18_O_2_	[M+H]^+^	207.1385	not identified	−2.4	-	-
**10**	8.89	169.0496	**169**; 141; 137; 125; 111; 110; 107; 79	C_8_H_8_O_4_	[M+H]^+^	169.05008	methyl 3,4-dihydroxybenzoate	−2.8	7	GNPS library
**11**	8.92	179.0703	179; **147**; 119	C_10_H_12_O_4_	[M+H-H_2_O]^+^	179.07081	dihydroferulic acid	−2.8	-	Manual inspection
**12**	8.93	389.2171	371; 227; **209**; 191; 163; 149; 125; 107; 85; 69	C_19_H_32_O_8_	[M+H]^+^	389.21754	4-[3-(β-D-glucopyranosyloxy)butyl]-4-hydroxy-3,5,5-trimethyl-2-cyclohexen-1-one	−1.1	13	GNPS library
**13**	9.30	371.2065	371; 353; **209**; 191; 125; 111	C_19_H_30_O_7_	[M+H]^+^	371.20697	ranuncoside	−1.3	-	Manual inspection
**14**	9.44	179.0704	179; **147**; 119	C_10_H_12_O_4_	[M+H-H_2_O]^+^	179.07081	dihydroisoferulic acid	−2.3	-	Manual inspection
**15**	9.62	211.0964	211; 193; 179; **170**; 147; 137; 123	C_11_H_14_O_4_	[M+H]^+^	211.09703	1-propanone, 1-(3,5-dimethoxy-4-hidroxyphenyl)	−3.0	-	Manual inspection
**16**	9.67	449.1069	449; 431; 413; 395; 383; 353, 339, **329**; 299	C_21_H_20_O_11_	[M+H]^+^	449.10838	orientin	−3.3	14	GNPS library
**17**	9.70	581.1502	**581**; 449; 431; 413; 383; 329; 299;	C_26_H_28_O_15_	[M+H]^+^	581.15064	2-*O*-β-D-xylopyranosylisoorientin	−0.8	13	GNPS library
**18**	9.73	177.0546	177; 163; 149; **145**; 135; 117; 89	C_10_H_10_O_4_	[M+H-H_2_O]^+^	177.05516	ferulic acid	−3.2	7	GNPS library
**19**	9.77	195.0653	195; **177**; 163; 145; 135; 117; 89	C_10_H_10_O_4_	[M+H]^+^	195.06573	ferulic acid	−2.2	7	GNPS library
**20**	9.85	611.1610	611; 449; 431; 413; 395; 383; 353; **329**; 311; 299; 287	C_27_H_30_O_16_	[M+H]^+^	611.1612	2-*O*-β-L-galactopyranosylorientin	−0.3	10	GNPS library
**21**	9.90	595.1657	**595**; 449; 413; 383; 353; 329; 299; 287	C_27_H_30_O_15_	[M+H]^+^	595.16629	isoorientin 2″-*O*-rhamnoside	−1.0	7	GNPS library
**22**	9.90	246.1490	**246**; 217; 177; 164; 137; 83; 55	C_15_H_19_NO_2_	[M+H]^+^	246.1494	not identified	−1.6	-	-
**23**	9.94	463.1236	463; 445; 427; 409; 397; 367; 353; 343; **313**	C_22_H_22_O_11_	[M+H]^+^	463.12403	swertiajaponin	−0.9	6	GNPS library
**24**	10.07	197.1174	197; **179**; 161; 135; 133; 107; 93	C_11_H_16_O_3_	[M+H]^+^	197.11776	loliolide	−1.8	7	GNPS library
**25**	10.09	565.1554	**565**; 433; 415; 397; 367; 337; 313; 283	C_26_H_28_O_14_	[M+H]^+^	565.15573	3′-hydroxypuerarin 2″-β-D-xyloside	−0.6	8	GNPS library
**26**	10.11	595.1658	595; 475; **433**; 415; 397; 337; 313; 271; 85	C_27_H_30_O_15_	[M+H]^+^	595.16629	isovitexin 2″-*O*-glucoside	−0.8	13	GNPS library
**27**	10.13	433.1127	**433**; 415; 397; 367; 349; 337; 313; 283	C_21_H_20_O_10_	[M+H]^+^	433.11347	vitexin	−1.8	9	GNPS library
**28**	10.18	579.1700	**579**; 433; 415; 397; 337; 313; 271; 217; 85	C_27_H_30_O_14_	[M+H]^+^	579.17138	vitexin 2″-*O*-rhamnoside	−2.4	13	GNPS library
**29**	10.32	433.1130	433; 415; 397; 367; 349; 337; **313**; 283	C_21_H_20_O_10_	[M+H]^+^	433.11347	isovitexin	−1.1	14	GNPS library
**30**	10.42	463.1232	463; 445; 427; 367; **343**; 313; 261; 217; 151; 96	C_22_H_22_O_11_	[M+H]^+^	463.12403	diosmetin 6-*C*-glucoside	−1.8	-	Manual inspection
**31**	10.43	593.1868	593; **447**; 429; 381; 351; 327; 297; 285	C_28_H_32_O_14_	[M+H]^+^	593.18703	acacetin 7-*O*-rutinoside	−0.4	4	GNPS library
**32**	10.53	447.1281	447; 429; 411; 393; 381; 351; 327; **297**; 285	C_22_H_22_O_10_	[M+H]^+^	447.12912	isoswertisin	−2.3	-	Manual inspection
**33**	10.60	209.0808	209; 194; 181; **177**; 149; 145; 121; 55	C_11_H_12_O_4_	[M+H]^+^	209.08138	sinapaldehyde	−2.8	6	GNPS library
**34**	10.65	387.2016	387; 355; 225; **207**; 189; 167; 149; 123	C_19_H_30_O_8_	[M+H]^+^	387.20189	roseoside	−0.7	-	Manual inspection
**35**	10.87	417.1184	**417**; 399; 381; 321; 297; 267;217; 167; 105	C_21_H_20_O_9_	[M+H]^+^	417.11855	pueranin	−0.4	17	GNPS library
**36**	10.88	183.0665	183; 155; **151**; 137; 124; 123; 111; 107; 93; 79	C_9_H_10_O_4_	[M+H]^+^	183.06573	methyl 3-hydroxy-4-methoxybenzoate	4.2	5	GNPS library
**37**	10.89	623.1972	623; 503; **461**; 425; 365; 341; 299; 127; 85	C_29_H_34_O_15_	[M+H]^+^	623.19759	embinoidin	−0.6	-	Manual inspection
**38**	10.94	433.1129	433; 415; 367; 337; 313; 283; **271**	C_21_H_20_O_10_	[M+H]^+^	433.11347	apigenin 7-*O*-glucoside	−1.3	-	Manual inspection
**39**	10.95	609.1814	609; 447; 429; 411; 381; 351; **327**; 297; 285	C_28_H_32_O_15_	[M+H]^+^	609.18194	swertisin-2″-*O*-glucoside	−0.9	9	GNPS library
**40**	10.96	593.1866	593; **447**; 429; 381; 327; 297; 285; 85	C_28_H_32_O_14_	[M+H]^+^	593.18703	swertisin-2″-*O*-rhamnoside	−0.7	4	GNPS library
**41**	11.07	447.1280	**447**; 429; 411; 381; 327; 297; 261; 162; 135; 96	C_22_H_22_O_10_	[M+H]^+^	447.12912	3′-methoxypuerarin	−2.5	13	GNPS library
**42**	11.20	461.1443	**461**; 341; 299	C_23_H_24_O_10_	[M+H]^+^	461.14477	7-(β-D-glucopyranosyloxy)-5-methoxy-2-(4-methoxyphenyl) 4H-1-benzopyran-4-one	−1.0	-	Manual inspection
**43**	11.38	211.1693	211; 193; 175; 151; 135; **109**; 95; 69	C_13_H_22_O_2_	[M+H]^+^	211.1698	not identified	−2.4	-	-
**44**	11.52	195.1016	195; 167; 163; **154**; 135; 107; 103; 91; 79	C_11_H_14_O_3_	[M+H]^+^	195.10211	6-methoxy eugenol	−2.6	6	GNPS library
**45**	11.68	447.1287	447; 429; 411; 393; 381; 351; 327; **297**; 285	C_22_H_22_O_10_	[M+H]^+^	447.12912	swertisin	−0.9	11	GNPS library
**46**	11.68	207.0652	207; 192; 179; **175**; 147; 119; 91	C_11_H_12_O_5_	[M+H-H_2_O]^+^	207.06573	*trans*-sinapic acid	−2.6	11	GNPS library
**47**	11.78	461.1466	461; 443; 425; 407; 395; 365; 351; 341; **311**; 159; 109	C_23_H_24_O_10_	[M+H]^+^	461.14477	embigenin	4.0	-	Manual inspection
**48**	11.81	179.0703	179; 151; **147**; 137; 123; **119**; 105; 91	C_10_H_10_O_3_	[M+H]^+^	179.07081	coniferaldehyde	−2.8	14	GNPS library
**49**	11.82	177.0545	177; 163; 149; **145**; 135; 117; 89	C_10_H_10_O_4_	[M+H-H_2_O]^+^	177.05516	isoferulic acid	−3.7	6	GNPS library
**50**	11.86	197.0811	197; 169; 151; **125**; 111; 93; 65	C_10_H_12_O_4_	[M+H]^+^	197.08138	ethyl vanillate	−1.4	7	GNPS library
**51**	11.89	489.1389	**489;** 471; 453; 411; 393; 327; 297; 121; 96	C_24_H_24_O_11_	[M+H]^+^	489.13968	2′-*O*-acetyl-7-*O*-methyl vitexin	−1.6	-	Manual inspection
**52**	12.19	207.0653	207; 192; 179; **175**; 147; 119; 91	C_11_H_12_O_5_	[M+H-H_2_O]^+^	207.06573	*cis*-sinapic acid	−2.1	10	GNPS library
**53**	12.33	447.1291	447; 429; 411; 393; 381; 351; 327; 297; **285**	C_22_H_22_O_10_	[M+H]^+^	447.12912	6-β-D-glucopyranosyl-7-hydroxy-2-(4-hydroxyphenyl)-5-methoxy-4H-1-benzopyran-4-one	0.0	10	GNPS library
**54**	12.45	271.0959	**271**; 229; 167; 131; 103	C_16_H_14_O_4_	[M+H]^+^	271.09703	alpinetin	−4.2	5	GNPS library
**55**	12.56	221.1172	221; 189; **165**; 153; 109; 69	C_13_H_16_O_3_	[M+H]^+^	221.11776	4-methoxy-3-(3′-methyl-2′-butenyl)-benzoic acid	−2.5	-	Manual inspection
**56**	12.56	207.1012	207; 165; **151**; 107; 69	C_12_H_14_O_3_	[M+H]^+^	207.10211	4-hydroxy-3-(3′-methyl-2′-butenyl)-benzoic acid	−4.4	-	Manual inspection
**57**	12.59	273.1124	273; 255; 245; 217; 169; 141; 133; **105**; 91	C_16_H_16_O_4_	[M+H]^+^	273.11268	2,6-dihydroxy-4-methoxydihydrochalcone	−1.0	8	GNPS library
**58**	12.61	237.1848	237; 219; 201; **191**; 159; 145; 135; 121; 95; 81	C_15_H_24_O_2_	[M+H]^+^	237.18545	bisabolene-1,4-endoperoxide	−2.7	10	GNPS library
**59**	12.71	275.2006	**275**; 257; 239; 161; 147; 133; 119; 105; 91;	C_18_H_28_O_3_	[M+H-H_2_O]^+^	275.2011	(10*E*,12*Z*,15*Z*)-9-oxooctadeca-10,12,15-trienoic acid	−1.8	8	GNPS library
**60**	12.73	293.2109	293; **275**; 257; 239; 189; 133; 107; 95; 81; 67	C_18_H_28_O_3_	[M+H]^+^	293.21166	not identified	−2.6	-	-
**61**	12.75	311.2216	311; 293; **275**; 257; 189; 121; 109; 95; 81; 67	C_18_H_32_O_5_	[M+H-H_2_O]^+^	311.22223	9,12,13-trihydroxyoctadeca-10,15-dienoic acid	−2.0	9	GNPS library
**62**	12.89	317.1020	**317**; 193; 185; 177; 167; 145;	C_17_H_16_O_6_	[M+H]^+^	317.10251	Eriodictyol-7,3′-dimethyl ether	−1.6	7	GNPS library
**63**	12.90	287.0909	**287**; 269; 167; 147; 119;	C_16_H_14_O_5_	[M+H]^+^	287.09194	sakuranetin	−3.6	7	GNPS library
**64**	12.97	274.1453	274; **201**; 171; 159; 143; 135; 115	C_16_H_19_NO_3_	[M+H]^+^	274.14431	piperlonguminine	3.6	8	GNPS library
**65**	12.98	257.0808	**257**; 212; 171; 153; 131	C_15_H_12_O_4_	[M+H]^+^	257.08138	pinocembrine	−2.3	6	GNPS library
**66**	13.17	285.1134	**285**; 243; **181;** 131; 91	C_17_H_16_O_4_	[M+H]^+^	285.11268	5,7-dimethoxyflavanone	2.5	5	GNPS library
**67**	13.22	286.1436	**286**; 201; 171; 143; 135; 112; 84	C_17_H_19_NO_3_	[M+H]^+^	286.14431	piperine	−2.5	12	GNPS library
**68**	13.25	273.1497	273; **217**; 199; 173; 159; 91; 69	C_17_H_20_O_3_	[M+H]^+^	273.14906	2,2-dimethyl-8-(3-methylbut-2-*en*-1-yl)-2H-chromene-6-carboxylic acid	2.3	-	Manual inspection
**69**	13.27	285.0758	**285**; 270; 242; 222; 139; 99; 68	C_16_H_12_O_5_	[M+H]^+^	285.07629	wogonin	−1.7	-	Manual inspection
**70**	13.48	287.1640	287; 231; 219; 175; 157; 105; **69**	C_18_H_22_O_3_	[M+H]^+^	287.16471	4-methoxy-3-(3-methylbut-2-en-1-yl)-5-(3-methylbuta-1,3-dien-1-yl)benzoic acid	−2.5	-	Manual inspection
**71**	13.50	275.2005	**275**; 239; 161; 147; 133; 119;105	C_18_H_28_O_3_	[M+H-H_2_O]^+^	275.2011	9-oxo-10,12,15-octadecatrienoic acid	−2.2	14	GNPS library
**72**	13.52	273.1118	273; 255; 223; 177; 133; 115; **105**; 91;	C_16_H_16_O_4_	[M+H]^+^	273.11268	2,4-dihydroxy-6-methoxydihydrochalcone	−3.2	8	GNPS library
**73**	13.53	289.1797	**289**; 257; 233; 221; 165; 153; 69	C_18_H_24_O_3_	[M+H]^+^	289.18036	(3′,7′-dimethyl-2′,6′-octadienyl)-4-methoxybenzoic acid	−2.3	-	Manual inspection
**74**	13.55	235.1328	235; 207; 189; **151**; 107; 69	C_14_H_18_O_3_	[M+H]^+^	235.13341	methyl 4-metoxy-3-(3′-methyl-2′-butenyl)benzoate	−2.6	-	Manual inspection
**75**	13.76	351.2528	**351**; 259; 241; 161; 147; 133; 93; 81; 67	C_21_H_34_O_4_	[M+H]^+^	351.25353	2,3-dihydroxypropyl-6,9,12,15-octadecatetraenoate	−2.1	-	Manual inspection
**76**	13.81	282.2791	**282**; 265;247; 163; 149; 135; 97; 83; 69; 55	C_18_H_35_NO	[M+H]^+^	282.27968	9-octadecenamide	−2.1	12	GNPS library
**77**	13.96	277.2160	277; 221; 163; 149; 135; 121; 107; 93; 79	C_18_H_30_O_3_	[M+H-H_2_O]^+^	277.21675	9-hydroxy-10,12,15-octadecatrienoic acid	−2.7	9	GNPS library
**78**	14.16	279.2318	279; 209; 173; 137; 123; 109; 95; **81**; 67	C_18_H_32_O_3_	[M+H-H_2_O]^+^	279.2324	9,10-epoxyoctadecenoic acid	−2.1	7	GNPS library
**79**	14.30	427.3890	427; **324**; 199; 71; 67;	C_25_H_50_N_2_O_3_	[M+H]^+^	427.38996	isostearamidopropyl betaine **	−2.2	-	Manual inspection
**80**	14.32	357.3000	357; 339; 283; **265**; 247; 149; 135; 121; 95; 81; 69	C_21_H_40_O_4_	[M+H]^+^	357.30048	monoolein	−1.3	12	GNPS library
**81**	14.41	338.3416	**338**; 321; 303; 135; 97; 83; 69; 55	C_22_H_43_NO	[M+H]^+^	338.34229	13-docosenamide	−2.0	12	GNPS library
**82**	14.55	305.2472	**305**; 163; 149; 135; 121; 107; 93; 79; 67; 55	C_20_H_34_O_3_	[M+H-H_2_O]^+^	305.24805	15-oxo-11(*Z*),13(*E*)-eicosadienoic acid	−2.8	9	GNPS library
**83**	14.73	372.3469	372; 354; 311; **106**; 88; 70	C_22_H_45_NO_3_	[M+H]^+^	372.34776	stearic diethanolamide **	−2.3	-	Manual inspection
**84**	14.74	293.2474	**293**; 261; 243; 137; 123; 109; 95; 81; 67	C_19_H_34_O_3_	[M+H-H_2_O]^+^	293.24805	13-hydroxy-9(*Z*),11(*E*)-octadecadienoic acid, methyl ester	−2.2	9	GNPS library
**85**	14.76	364.3574	**364**; 282; 247; 121; 97; 83; 69; 55	C_24_H_45_NO	[M+H]^+^	364.35794	pipzorine	−1.5	-	Manual inspection
**86**	14.82	353.2683	353; **261**; 243; 233; 173; 121; 109; 95; 81	C_21_H_36_O_4_	[M+H]^+^	353.26918	monolinolenin	−2.5	10	GNPS library
**87**	14.94	470.4203	470; 288; **270**; 227; 106; 88	C_28_H_55_NO_4_	[M+H]^+^	470.42093	not identified	−1.3	-	-
**88**	15.31	355.2840	355; 337; **263**; 245; 161; 121; 109; 95; 81; 67	C_21_H_38_O_4_	[M+H]^+^	355.28483	monolinolein	−2.3	8	GNPS library
**89**	15.52	307.2627	**307**; 261; 243; 137; 123; 109; 95; 81; 67	C_20_H_34_O_2_	[M+H]^+^	307.2637	9(*Z*),11(*E*),13(*E*)-octadecatrienoic acid, ethyl ester	−3.3	6	GNPS library

* In bold: base peak; ** possible contaminant.

## 3. Materials and Methods

### 3.1. Plant Material

Adult specimens of *Piper aduncum* L., in the reproductive stage, were collected in the Agroecological Cultivation System at the Socio-Environmental Responsibility Center of the Rio de Janeiro Botanical Garden Research Institute, Brazil, (S22°58′0″ W43°13′43″). Leaves (1100 g), stems (950 g), and reproductive organs (inflorescences and infructescences, 100 g) were harvested for the experiments. The material was authenticated by Dr. Elsie Franklin Guimarães and Dr. George Azevedo Queiroz, both from the Rio de Janeiro Botanical Garden Research Institute, where a voucher specimen was deposited with the number RB01426180. The studies were registered in the National System for Management of Genetic Heritage and Associated Traditional Knowledge (SisGen) under the number AE4E953. The plants were farmed in full sun, in plots arranged in 1.5 × 1.5 m spaces, with a base fertilizer application of 40 kg/ha. Irrigation was performed daily, and the soil was maintained in a field capacity condition. For more details about this Agroecological Cultivation System see [16].

### 3.2. Extract Preparation and Column Chromatography of the Bioactive Partition

The plant material was crushed and dried in an air-circulated oven at 40 °C until reaching a constant weight, resulting in 292.97 g of leaves, 223.03 g of stems, and 16.42 g of reproductive organs (inflorescences/infructescences). The dried materials were ground into powder using a knife mill and extracted by static maceration in 70% (*v*/*v*) ethanol/ultrapure water, with solvent exchange every three days. The ethanolic extracts from leaves (PAEEL), stems (PAEES), and inflorescence/infructescence (PAEEI) were concentrated under reduced pressure using a rotatory evaporator with a heating bath (Fisatom, São Paulo, Brazil), equipped with a V-100 Buchi vacuum pump (Buchi, Flawil, Switzerland), resulting in 77.63 g, 30.94 g, and 5.20 g, respectively. Then, crude extracts were resuspended in 70% (*v*/*v*) methanol/ultrapure water and subjected to liquid–liquid partitioning with *n*-hexane, dichloromethane, ethyl acetate, and butanol (800 mL each). The aqueous residue was lyophilized and not used in this procedure. The solvent was evaporated under reduced pressure, yielding the following partitions: (a) leaves—PAHPL (*n*-hexane, 10.40 g), PADPL (dichloromethane, 4.65 g), PAEPL (ethyl acetate, 1.61 g), and PABPL (butanol, 6.14 g); (b) stems—PAHPS (*n*-hexane, 1.22 g), PADPS (dichloromethane, 0.50 g), PAEPS (ethyl acetate, 0.62 g), and PABPS (butanol, 1.31 g); and (c) inflorescences/infructescences—PAHPI (*n*-hexane, 0.53 g), PADPI (dichloromethane, 0.18 g), PAEPI (ethyl acetate, 0.34 g), and PABPI (butanol, 0.30 g).

As the ethyl acetate partition from leaves (PAEPL, 1.61 g) showed the highest activity in the antimycobacterial assay, for this reason, it was submitted to a chromatographic column for pre-purification. A total of 200 mg of the partition was subjected to open glass column chromatography (1000 mm × 20 mm), using Sephadex^®^ LH-20 (Sigma-Aldrich, São Paulo, SP, Brazil) as the stationary phase and methanol as the eluent. This procedure was repeated 4 times. The chromatographic separation resulted in 5 fractions, which were referred to as SFR1 (6.4 mg), SFR2 (61.0 mg), SFR3 (97.1 mg), SFR4 (45.5 mg), and SFR5 (84.9 mg). All solvents used were spectroscopic-grade and were obtained from Sigma-Aldrich, Brazil.

The fractions SFR1–SFR5 were analyzed via TLC and UHPLC-HRMS/MS, and the data from MS were processed using the online GNPS platform.

TLC evaluation (prepared using silica gel plates, RF254 nm, Sigma-Aldrich, Brazil, and a mobile phase composed of mixtures of hexane, ethyl acetate, and methanol in different proportions) was performed under ultraviolet light, as well as with a 5% sulfuric acid solution in ethanol (both from Sigma-Aldrich, Brazil), and subsequent heating for compound visualization.

### 3.3. Analysis by Ultra-High-Performance Liquid Chromatography Coupled with High-Resolution Mass Spectrometry in Tandem (UHPLC-HRMS/MS)

Crude extracts and partitions (10 mg/mL) were subjected to exploratory analysis by UHPLC-HRMS/MS using an UltiMate 3000 UHPLC system (Thermo Fisher Scientific, Waltham, MA, USA) coupled to an Orbitrap Q Exactive Plus mass spectrometer (Thermo Scientific, Waltham, MA, USA) with an electrospray ionization source. A Waters^®^ Acquity UPLC BEH C18 chromatographic column (100 mm × 2.1 mm I.D. × 1.7, μm particle size) (Waters, Milford, MA, USA) was employed. Mobile phases A and B were used: A—ultrapure water with 0.1% formic acid, and B—methanol with 0.1% formic acid. The gradient elution was as follows: 0.0–4.0 min 15% B; 4.0 min 15% B; 10.0 min 95% B, 10.0–12.0 min 95% B; 13.0 min 15% B; 13.0–17.0 min 15% B. The flow rate was set at 0.35 mL/min, with a 5 μL injection volume and a column oven temperature of 40 °C. As parameters of the ionization source, sheath gas and auxiliary gas were used at 50 and 15 arbitrary units, respectively. The spray voltage was + or –3600 V, the S-lens voltage was 50 V, the capillary temperature was 320 °C, and the source temperature was 400 °C. Data acquisition was performed in Full Scan mode (total ion scan) in the *m*/*z* range of 100–1000; positive ionization mode, with a resolution of 35,000 (FWHM), AGC 1 × 10^6^, and IT 100 ms, combined with a data-dependent acquisition experiment (ddMS^2^ top3) at 17,500 (FWHM), AGC 1 × 10^5^, and IT 50 ms; NCE 15–35; and an isolation window of 1.2 Da.

### 3.4. Processing of UHPLC-HRMS/MS Data by Molecular Network

The UHPLC-HRMS/MS data obtained in the raw format of the positive ionization mode were converted to the mzXML format using MSConvert software at version 3 (Proteowizard Software Foundation, Palo Alto, CA, USA). The data were processed using MZmine 2.53 [66], with 5.0 × 10^6^ as the noise level intensity for MS^1^ data and 1.5 × 10^5^ for MS^2^ data, 0.02 as the *m*/*z* tolerance, and 0.04 as the minimum time span. The Wavelets—ADAP algorithm was used in the chromatogram deconvolution step, and a 0.2 minimum retention time (Rt) tolerance was used in the chromatogram alignment. The processed data were then exported and submitted for analysis on the online platform GNPS (Global Natural Product Social Molecular Network, https://gnps.ucsd.edu (accessed on 04 March 2024) [67]) using the Feature-Based Molecular Networking (FBMN) workflow. For FBMN, the precursor and fragment ion mass tolerance were both set to 0.02 Da, and the edges were filtered to have a cosine score above 0.75 and more than 4 matched peaks. The molecular networks created in the GNPS were imported and visualized using Cytoscape software (Version 3.8.0).

### 3.5. Biological Assay

#### 3.5.1. *Mycobacterium tuberculosis* H37Rv Growth

The virulent standard strain of *M. tuberculosis* H37Rv (ATCC, 27294) was cultured in 7H9 medium (BD Difco, Cockeysville, MD, USA) supplemented with 10% albumin, dextrose, catalase (ADC) (BD BBL), and 0.05% tween 80%. The cultures were maintained in an incubator (Thermo Fisher Scientific, Waltham, MA, USA) at 37 °C, under biosecurity level 3 containment conditions until the exponential growth phase.

#### 3.5.2. Growth Inhibition Assay

The samples were assessed for their antimycobacterial activity using the tetrazolium salt assay in a 96-well microplate at concentrations of 32, 64, and 128 μg/mL. For this assay, the *M. tuberculosis* H37Rv suspension was plated (1 × 10^6^ CFU/well) and incubated in the presence of samples or rifampicin. The sealed plate was incubated at 37 °C and 5% CO_2_ for 5 days. After this period, the bacterial cultures were incubated for 3 h with tetrazolium salt 3-[4,5-dimethylthiazol-2-yl]-2,5-diphenyltetrazolium (MTT) solution (5 mg/mL) in sterile phosphate-buffered saline (PBS) and then lysis buffer (20% *w*/*v* sodium dodecyl sulfate (SDS)/50% dimethylformamide (DMF) in distilled water, pH 4.7) was added overnight. The microplate was read in a spectrophotometer at 570 nm [67]. Rifampicin treatment (0.008, 0.04, 0.2, and 1 µg/mL) in wells containing only bacilli was used as a positive control (C+) for antimycobacterial activity. Wells containing bacilli without treatment were used as a negative control (C−). The percentage of the inhibition of mycobacterial growth was calculated using Equation (1) where O.D. = optical density.

100 − (O.D.sample − O.D.C+) × 100/(O.D.C− − O.D.C+)(1)

## 4. Conclusions

This study on the chemical composition of the ethyl acetate partition (active against *M. tuberculosis*) from the ethanolic extract of *P. aduncum* leaves provides valuable insights into the chemistry of a Piperaceae species widely used in traditional medicine. This is the first investigation into the chemistry of this species under agroecological cultivation. The chemistry of this cultivated specimen is notable, particularly for free and glycosylated flavonoids, benzoic acid derivatives (including prenylated ones), glycosides, free fatty acids, and glycerol-esterified fatty acids. The chemistry of this cultivated specimen is quite similar to those previously described for specimens collected in the wild, except for some annotated substances that had not been previously reported for the species. Therefore, it can be concluded that the cultivation of the species does not substantially alter its chemistry and can be undertaken for harnessing the medicinal potential offered by *P. aduncum* without posing risks to the native population of this Piperaceae species.

## Figures and Tables

**Table 1:** Inhibitory effect of the extracts and partitions of *Piper aduncum* L. against *Mycobacterium tuberculosis* H37RV.

Sample	MIC_50_ (µg/mL)
PAEEL	121.6 ± 1.04
PAHPL	60.65 ± 1.05
PADPL	39.74 ± 1.02
**PAEPL**	**27.98 ± 1.01**
PABPL	>128
PAEES	>128
**PAHPS**	**29.74 ± 1.03**
PADPS	59.05 ± 1.04
PAEPS	145.40 ± 1.05
PABPS	>128
PAEEI	>128
PAHPI	48.61 ± 1.06
PADPI	>128
PAEPI	>128 NC
PABPI	>128 NC

Legend: PAEEL—Ethanolic extract from leaves; PAHPL—Hexane partition from leaves’ ethanolic extract; PADPL—Dichloromethane partition from leaves’ ethanolic extract; PAEPL—Ethyl acetate partition from leaves’ ethanolic extract; PABPL—Butanol partition from leaves’ ethanolic extract; PAEES—Ethanolic extract from stems; PAHPS—Hexane partition from stems’ ethanolic extract; PADPS—Dichloromethane partition from stems’ ethanolic extract; PAEPS—Ethyl acetate partition from stems’ ethanolic extract; PABPS—Butanol partition from stems’ ethanolic extract; PAEEI—Ethanolic extract from inflorescences; PAHPI—Hexane partition from inflorescences’ ethanolic extract; PADPI—Dichloromethane partition from inflorescences’ ethanolic extract; PAEPI—Ethyl acetate partition from inflorescences’ ethanolic extract; PABPI—Butanol partition from inflorescences’ ethanolic extract.

## Data Availability

The complete molecular network and other parameters used for its construction are publicly available at the GNPS job link https://gnps.ucsd.edu/ProteoSAFe/status.jsp?task=f084b8f4e5834fc984b37d04141c7a81 (accessed on 4 March 2024).

## Supplementary Materials

The following supporting information can be downloaded at https://www.mdpi.com/article/10.3390/molecules29081690/s1: Figure S1: Molecular family monoglycosylated flavonoids; Figure S2: Molecular family diglycosylated flavonoids; Figure S3: Molecular family non-glycosylated flavonoids; Figure S4: Molecular family of cinnamic acid derivatives; Figure S5: Molecular family of prenylated acid derivatives and chromenes; Figure S6: Molecular family of derivatives of methoxibenzoic acid, phenylpropanoids, and C_6-_C_3_ derivative; Figure S7: Molecular family of glycosides; Figure S8: Molecular family of glyceride derivatives; Figure S9: Molecular family of fatty acids; Figure S10: Compounds annotated in clusters of two or three nodes, or in the form of self-loops in the molecular networks of the bioactive ethyl acetate partition from the ethanolic extract of *Piper aduncum* L.; and Figure S11: Compounds annotated in clusters of two or three nodes, or in the form of self-loops in the molecular networks.
