# Supplementary material for: UHPLC-HRMS/MS Chemical Fingerprinting of the Bioactive Partition from Cultivated Piper aduncum L
Source: Molecules. 2024 Apr 9;29(8):1690. doi: 10.3390/molecules29081690 (PMC11051932; doi:10.3390/molecules29081690)
Supplement: Supplementary file 1 [file molecules-29-01690-s001.zip › molecules-2937355-supplementary.pdf]

Supplementary Material

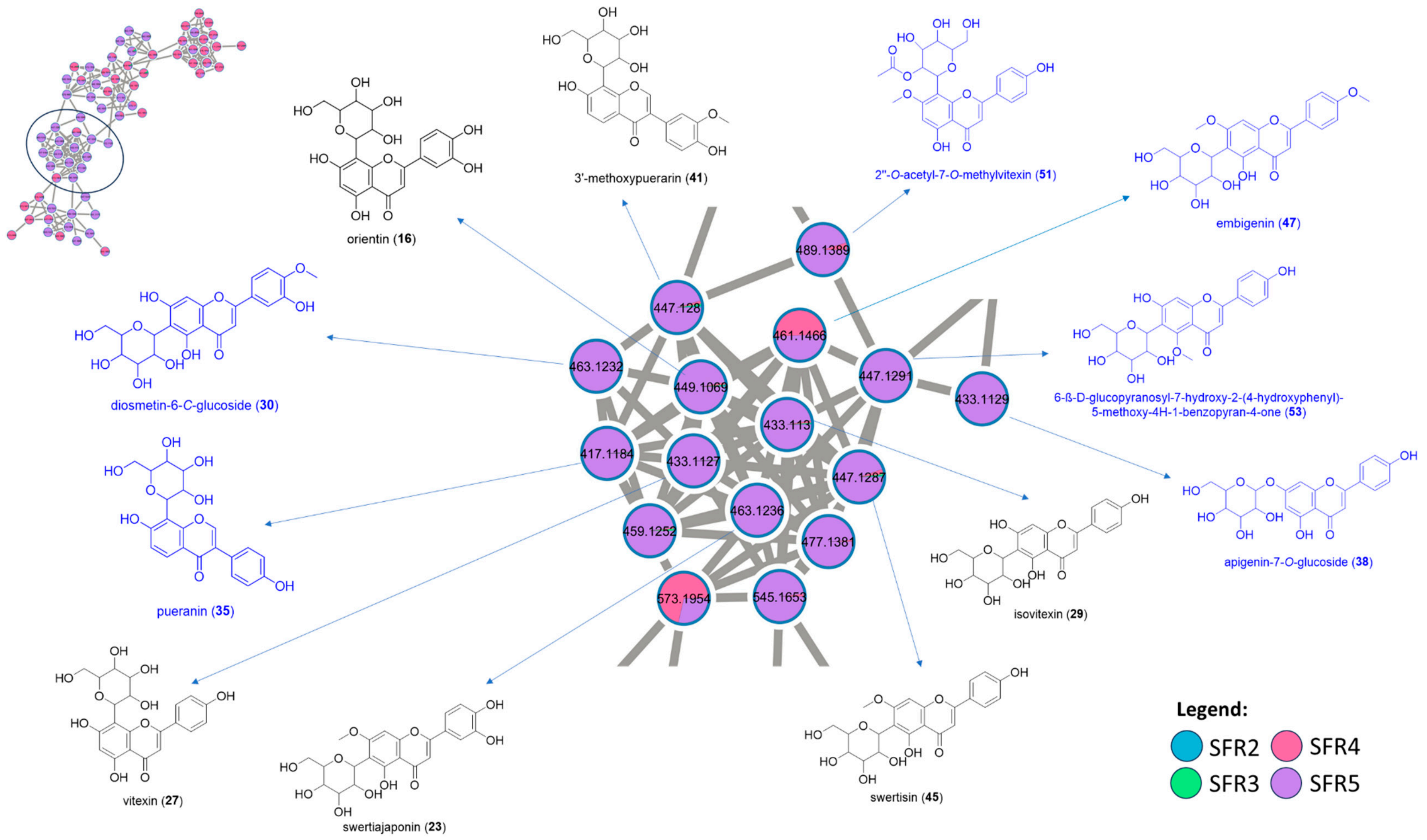

Figure S1 - Molecular family of monoglycosylated flavonoids. SFR2 – SFR5 = fractions 2 to 5 obtained by Sephadex LH-20 column chromatography of the bioactive ethyl acetate partition from the ethanolic extract of *Piper aduncum* L. In blue, substances annotated through manual inspection of the data, and in black, substances annotated by comparison with the GNPS library. All substances were proposed based on fragmentations that gave rise to characteristic ions and fragmentation profiles.

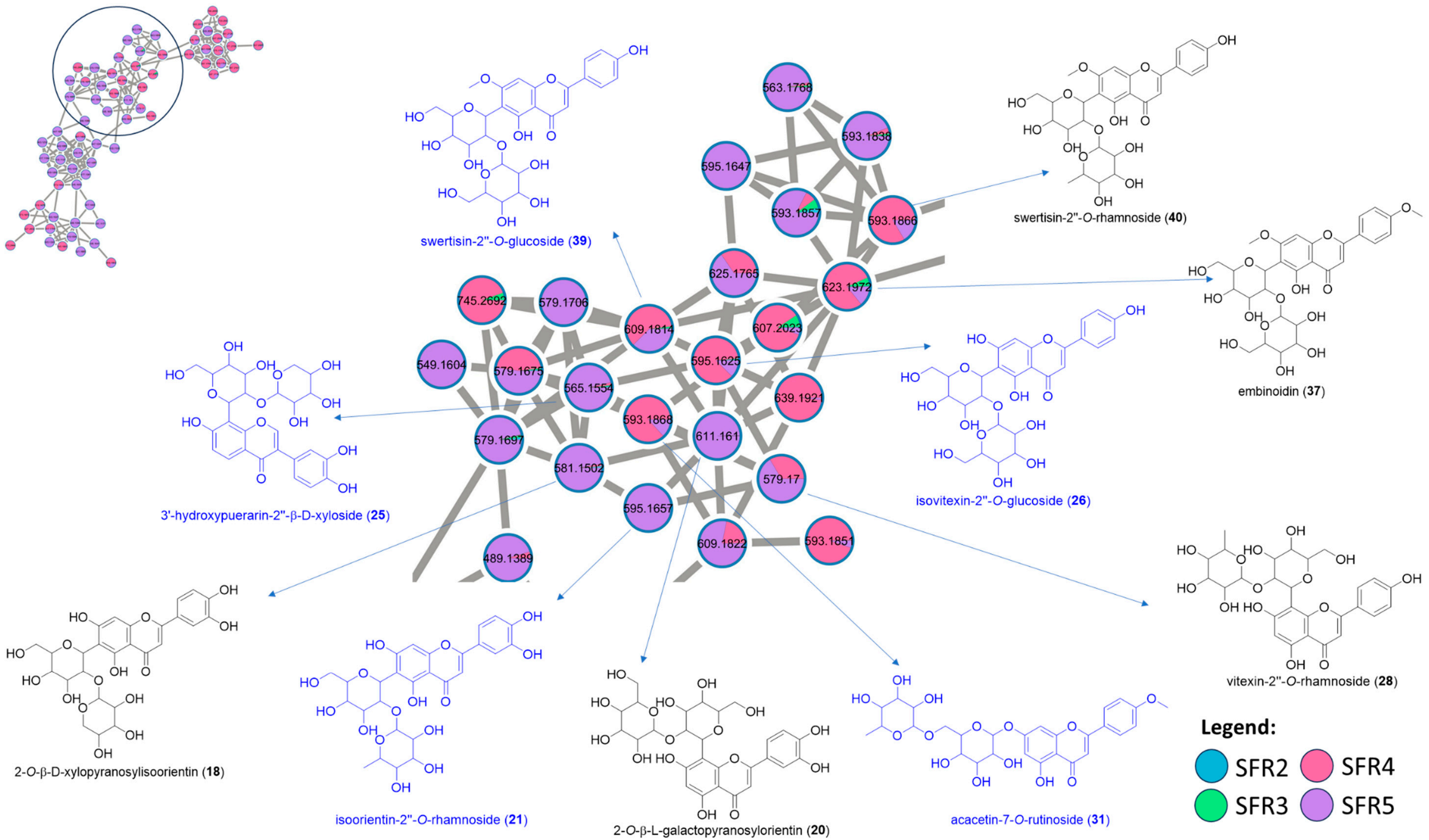

Figure S2 - Molecular family of diglycosylated flavonoids. SFR2 – SFR5 = fractions 2 to 5 obtained by Sephadex LH-20 column chromatography of the bioactive ethyl acetate partition from the ethanolic extract of *Piper aduncum* L. In blue, substances annotated through manual inspection of the data, and in black, substances annotated by comparison with the GNPS library. All substances were proposed based on fragmentations that gave rise to characteristic ions and fragmentation profiles.

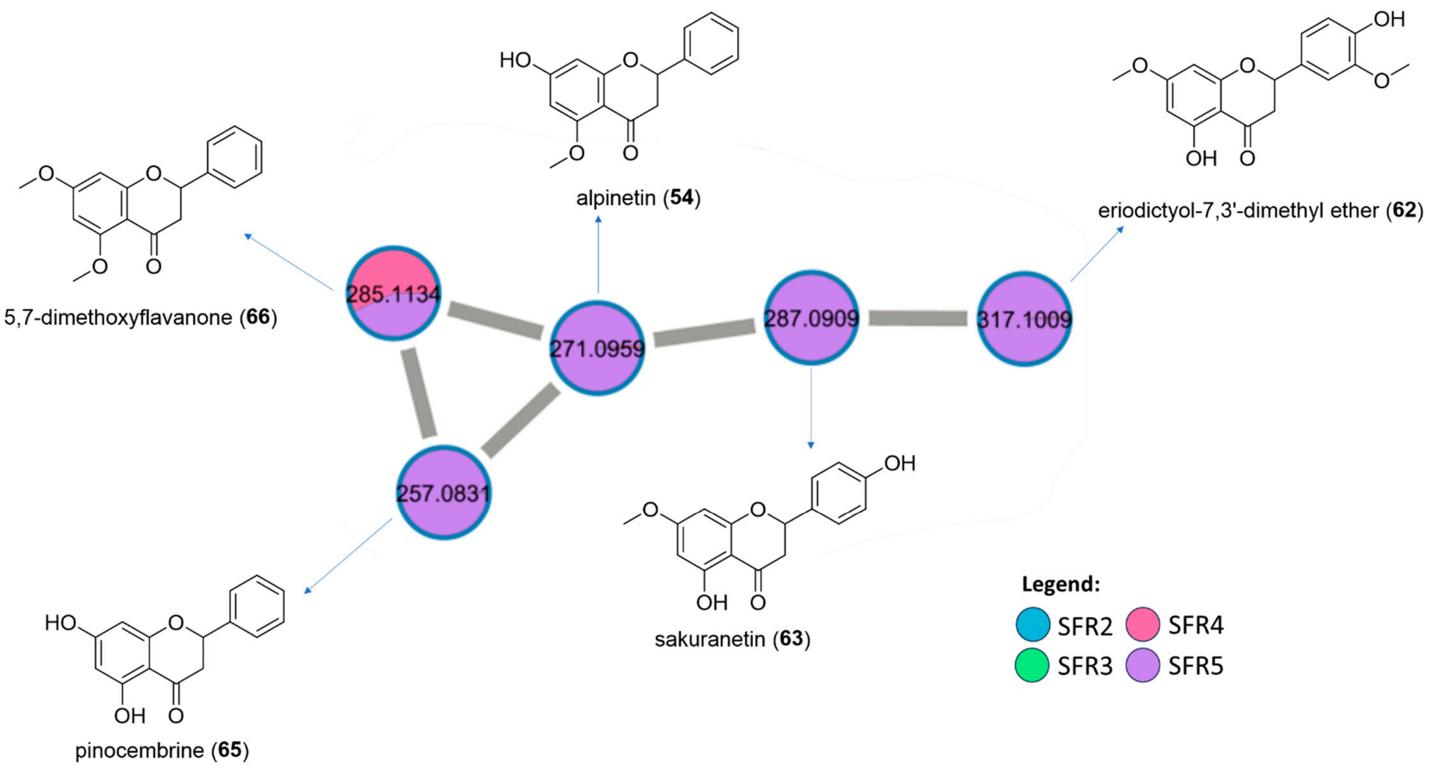

Figure S3 - Molecular family of non-glycosylated flavonoids. SFR2 – SFR5 = fractions 2 to 5 obtained by Sephadex LH-20 column chromatography of the bioactive ethyl acetate partition from the ethanolic extract of *Piper aduncum* L. All substances were annotated by comparison with the GNPS library. These substances were confirmed by fragmentations that gave rise to characteristic ions and the fragmentation profile.

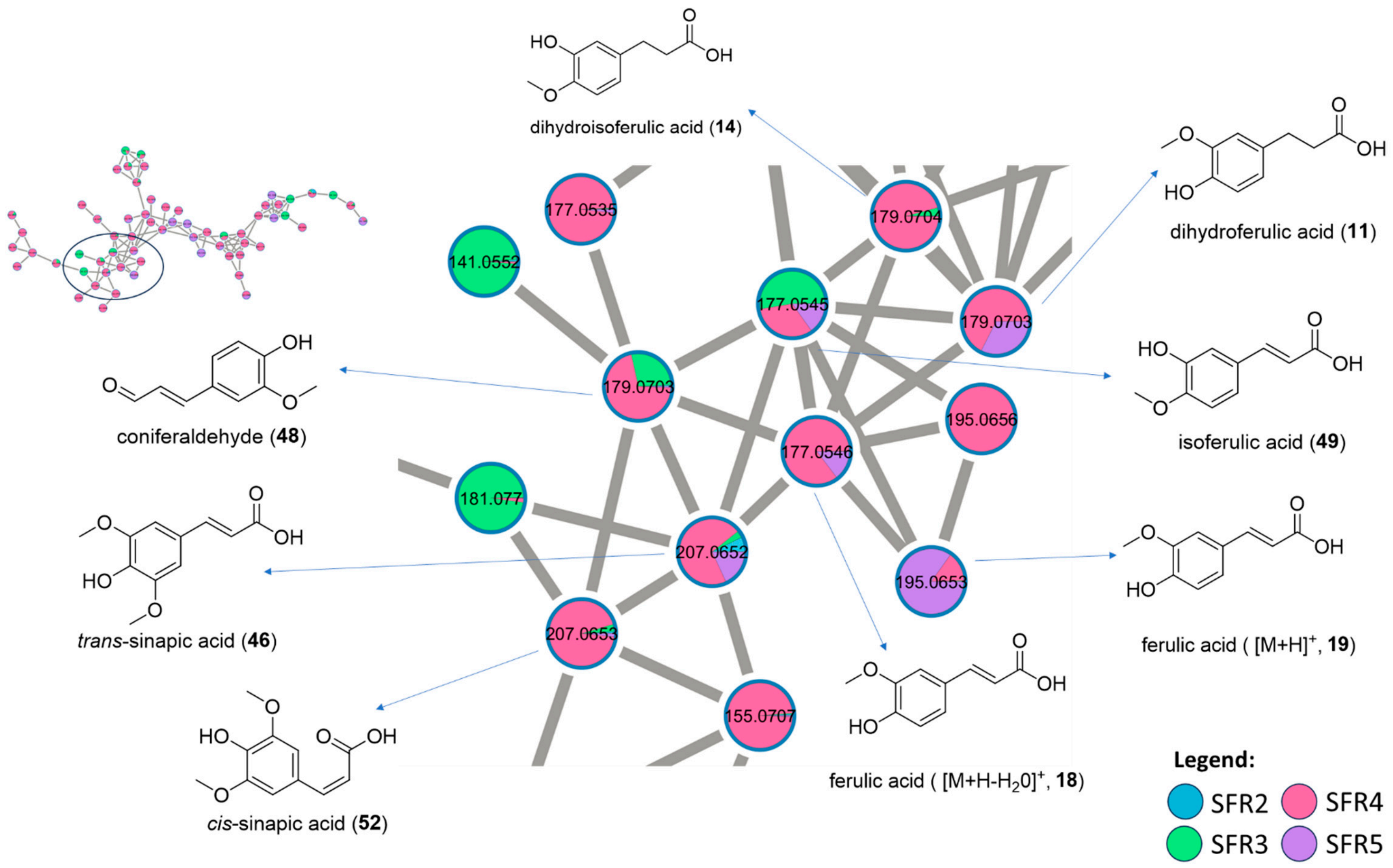

Figure S4 – Molecular family of cinnamic acid derivatives. SFR2 – SFR5 = fractions 2 to 5 obtained by Sephadex LH-20 column chromatography of the bioactive ethyl acetate partition from the ethanolic extract of *Piper aduncum* L. All substances were annotated by comparison with the GNPS library. These substances were confirmed by fragmentations that gave rise to characteristic ions and the fragmentation profile.

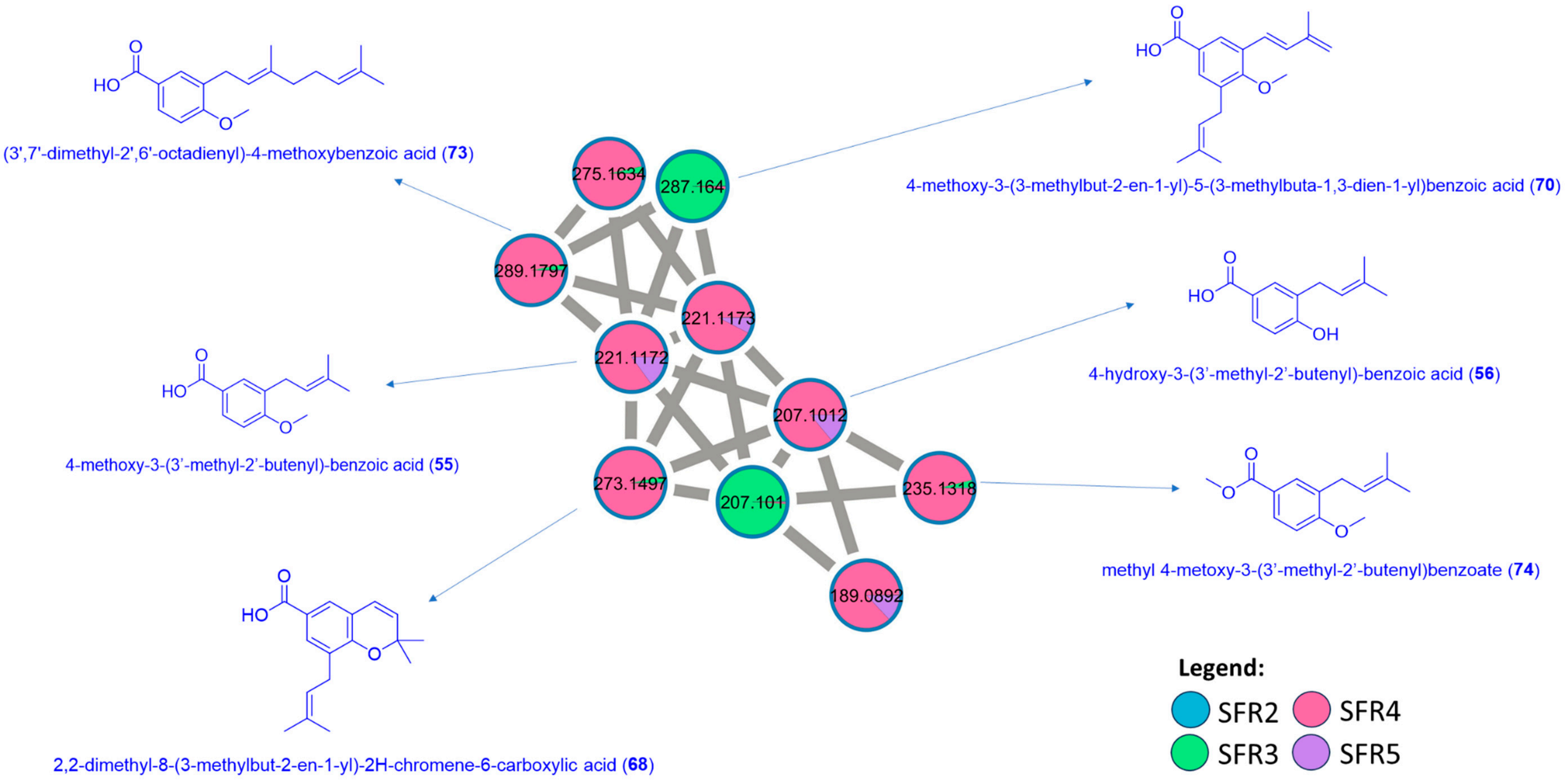

Figure S5 - Molecular family of prenylated benzoic acid derivatives and chromene **68**. SFR2 – SFR5 = fractions 2 to 5 obtained by Sephadex LH-20 column chromatography of the bioactive ethyl acetate partition from the ethanolic extract of *Piper aduncum* L. In blue, substances annotated by manual inspection of the data. All substances were proposed based on fragmentations that gave rise to characteristic ions and fragmentation profile.

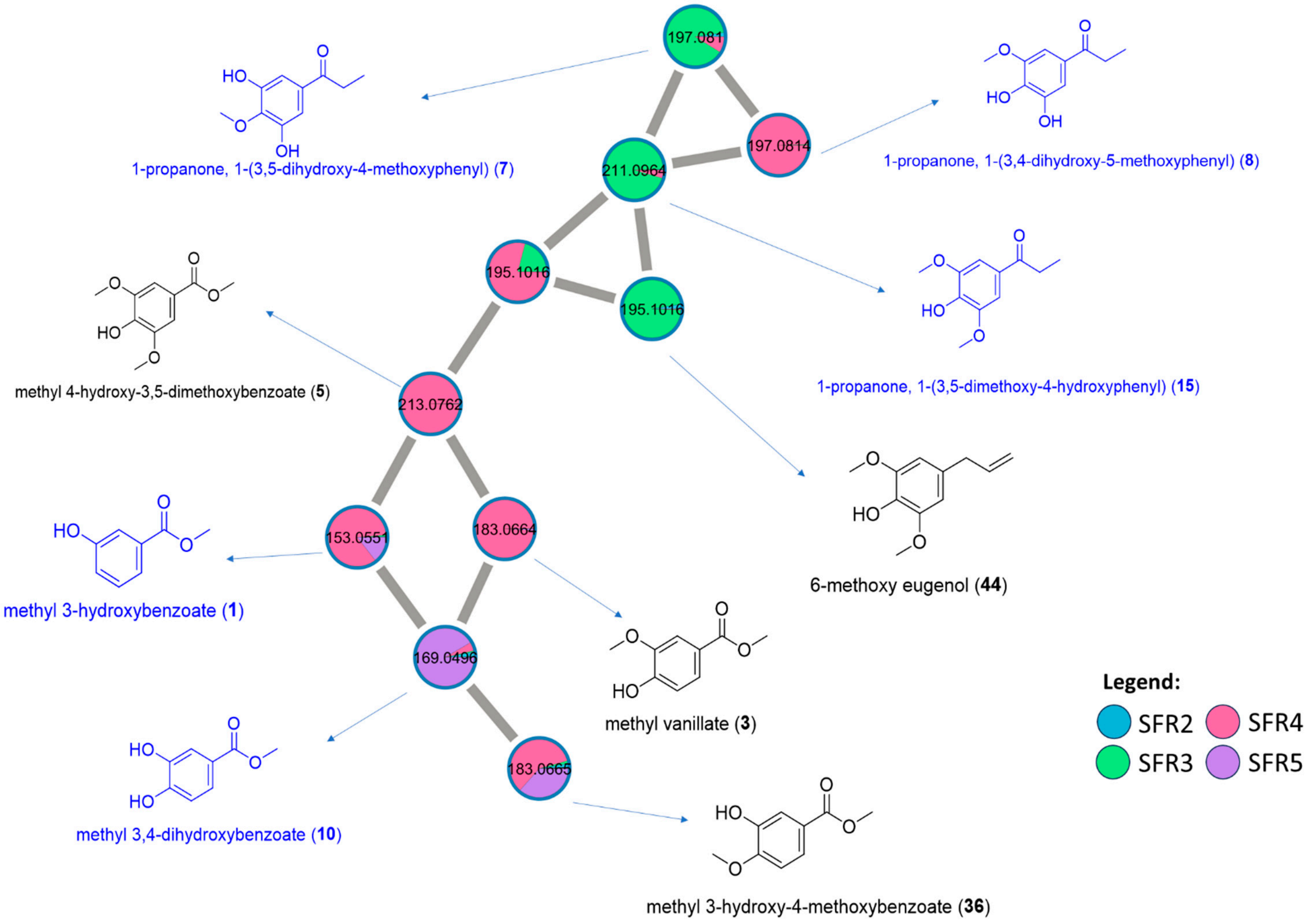

Figure S6 - Molecular family of derivatives of methoxibenzoic acid, phenylpropanoids **44**, and C_6-C_3 derivatives (7, 8, and 15). SFR2 – SFR5 = fractions 2 to 5 obtained by Sephadex LH-20 column chromatography of the bioactive ethyl acetate partition from the ethanolic extract of *Piper aduncum* L. In blue, substances annotated by manual inspection of the data, and in black, substances annotated by comparison with the GNPS library. All substances were proposed based on fragmentations that gave rise to characteristic ions and fragmentation profile.

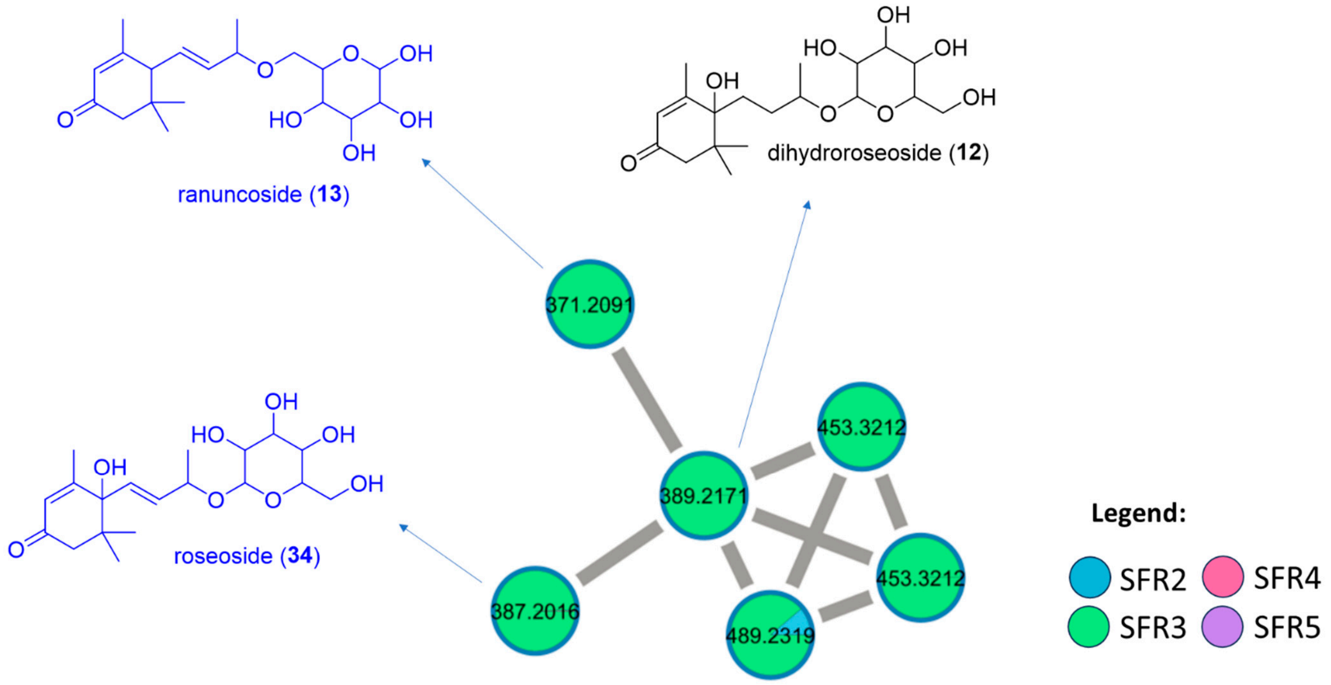

Figure S7 - Molecular family of glycosides. SFR2 – SFR5 = fractions 2 to 5 obtained by Sephadex LH-20 column chromatography of the bioactive ethyl acetate partition from the ethanolic extract of *Piper aduncum* L. In blue, substances annotated by manual inspection of the data, and in black, substances annotated by comparison with the GNPS library. All substances were proposed based on fragmentations that gave rise to characteristic ions and fragmentation profile.

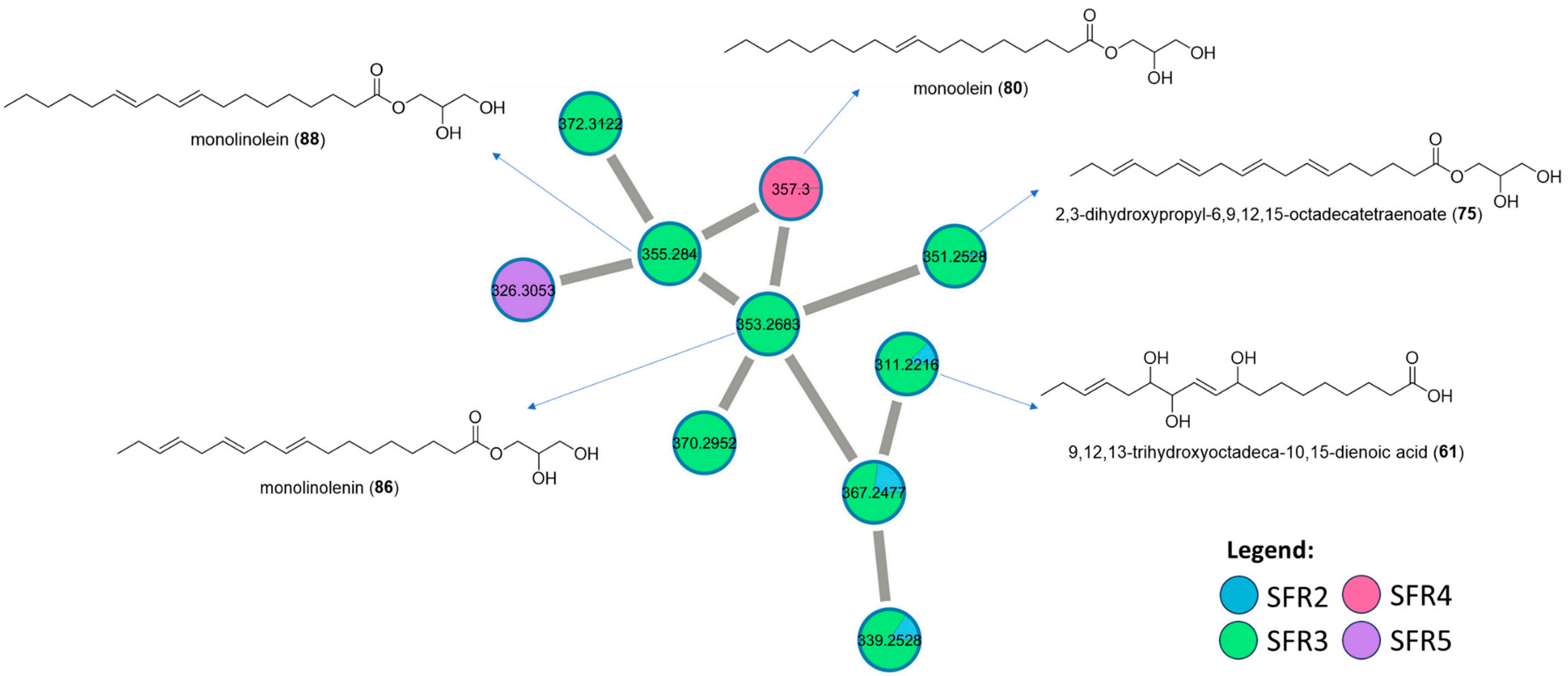

Figure S8 - Molecular family of glyceride derivatives. SFR2 – SFR5 = fractions 2 to 5 obtained by Sephadex LH-20 column chromatography of the bioactive ethyl acetate partition from the ethanolic extract of *Piper aduncum* L. All substances were annotated by comparison with the GNPS library. All substances were proposed based on fragmentations that gave rise to characteristic ions and the fragmentation profile.

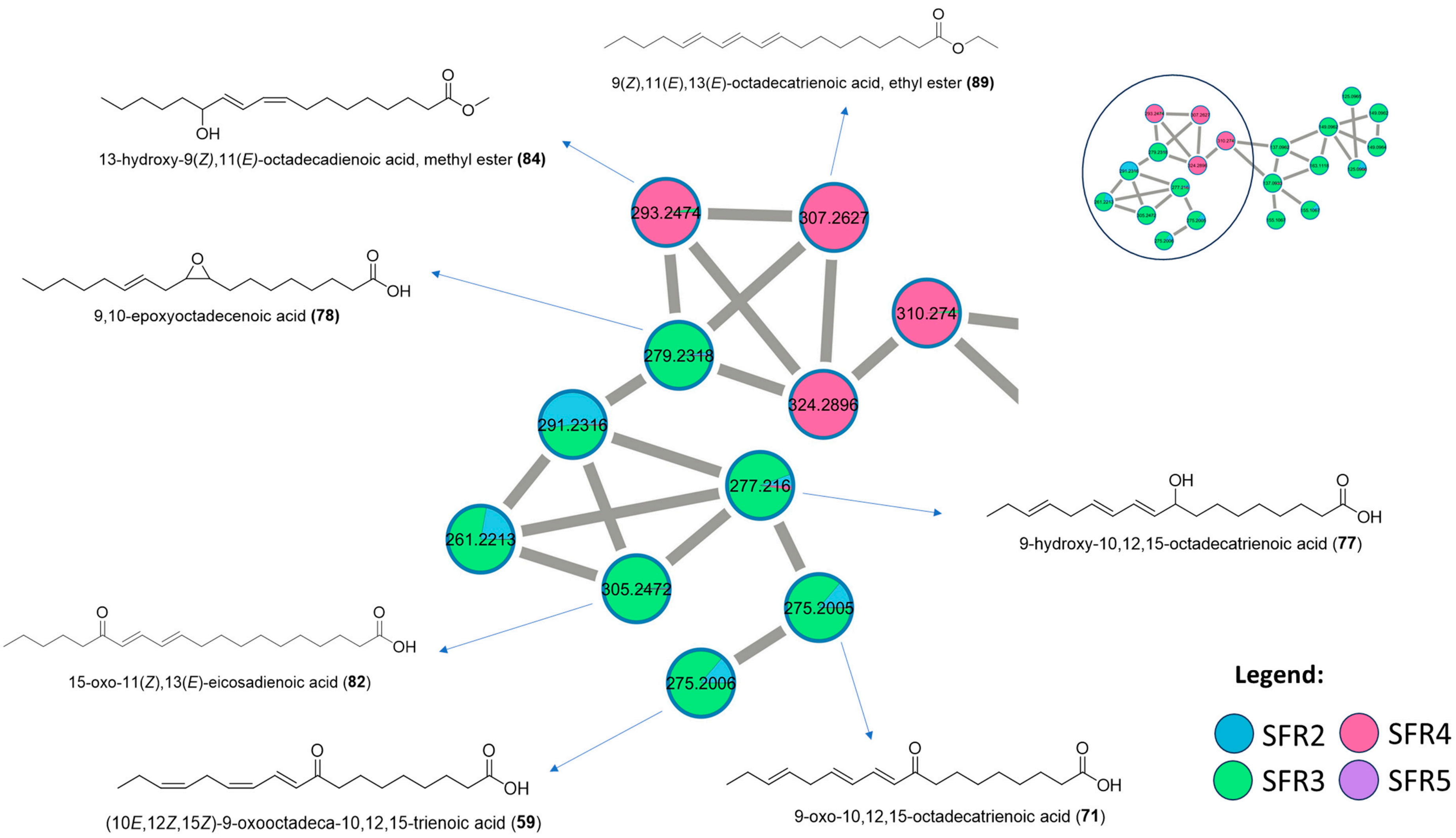

Figure S9 - Molecular family of fatty acids. SFR2 – SFR5 = fractions 2 to 5 obtained by Sephadex LH-20 column chromatography of the bioactive ethyl acetate partition from the ethanolic extract of *Piper aduncum* L. All substances were annotated by comparison with the GNPS library. All substances were proposed based on fragmentations that gave rise to characteristic ions and the fragmentation profile.

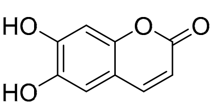

esculetin (**2**)

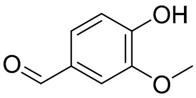

vanillin (**6**)

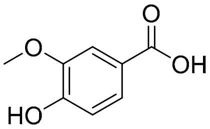

vanillic acid (**4**)

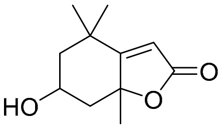

loliolide (**24**)

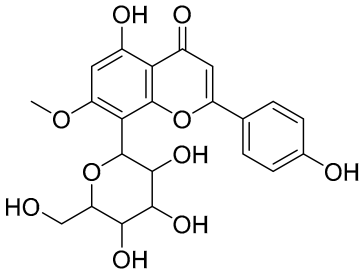

isoswertisin (**32**)

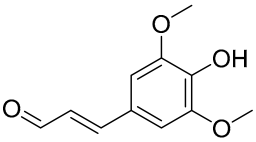

sinapaldehyde (**33**)

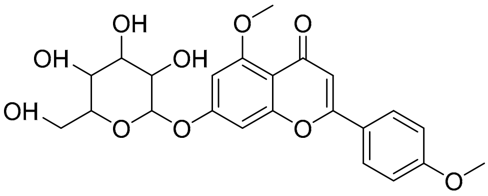

7-(β-D-glucopyranosyloxy)-5-methoxy-2-(4-methoxyphenyl)-4H-1-benzopyran-4-one (**42**)

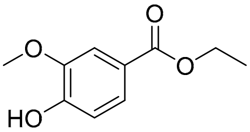

ethyl vanillate (**50**)

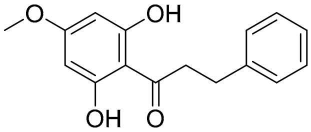

2,6-dihydroxy-4-methoxydihydrochalcone (**57**)

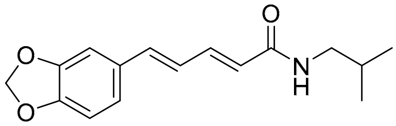

piperlonguminine (**64**)

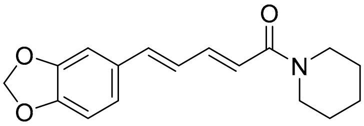

piperine (**67**)

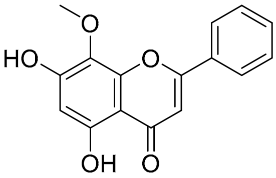

wogonin (**69**)

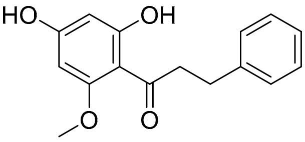

2,4-dihydroxy-6-methoxydihydrochalcone (**72**)

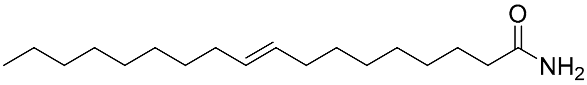

9-octadecenamide (**76**)

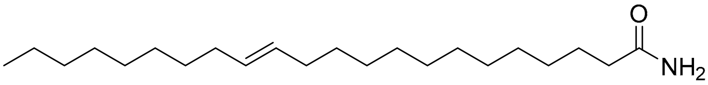

13-docosenamide (**81**)

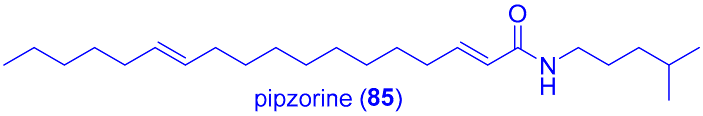

pipzorine (**85**)

Figure S10: Compounds annotated in clusters of two or three nodes, or in the form of self-loops in the molecular networks of the bioactive ethyl acetate partition from the ethanolic extract of *Piper aduncum* L. In blue, the substances annotated through manual inspection of the data, and in black, the substances annotated by comparison with the GNPS library. All substances were proposed based on fragmentations that gave rise to characteristic ions and fragmentation profiles.

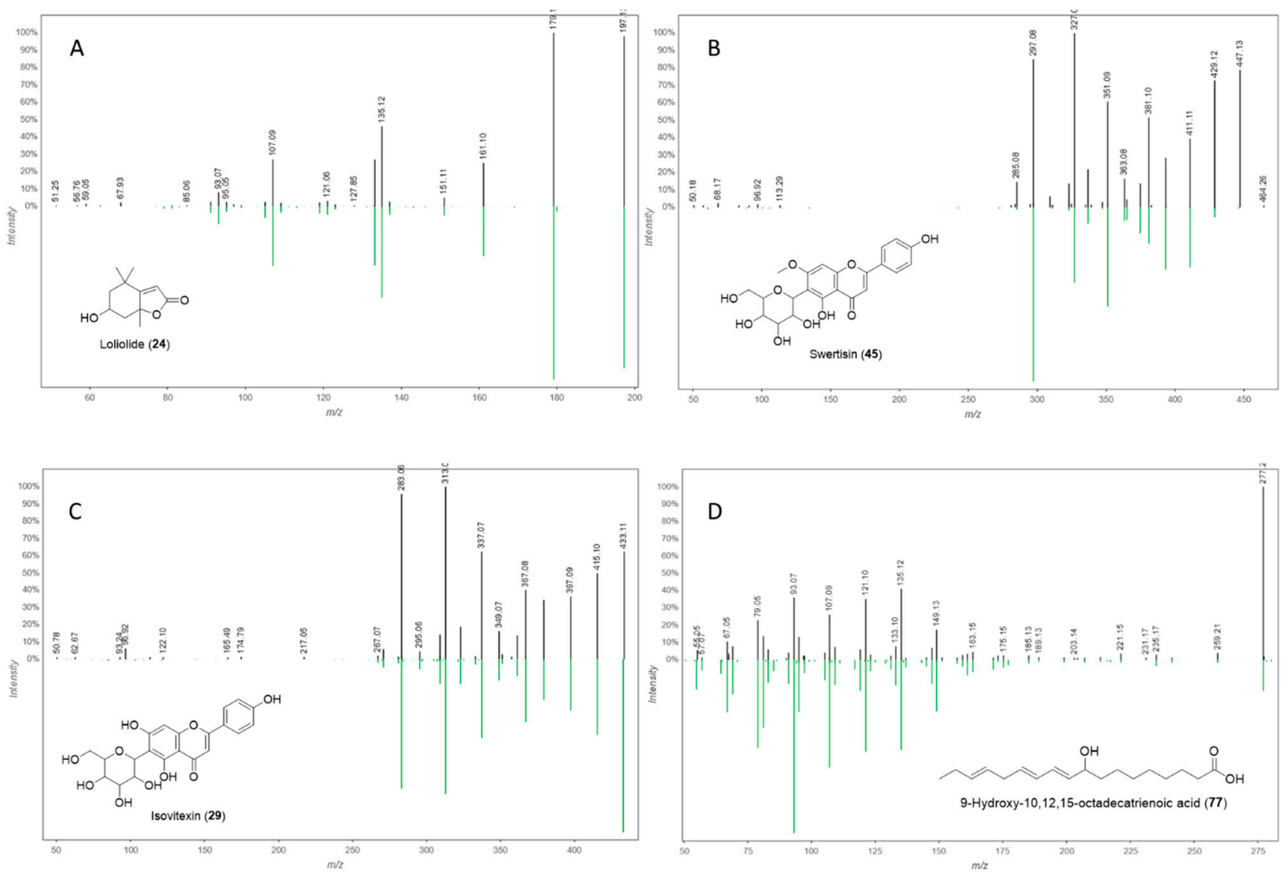

Figure S11 – MS/MS comparison of some main constituents of the bioactive ethyl acetate partition from the ethanolic extract of *Piper aduncum* L. A = loliolide; B = swertisin; C = isovitexin; D = 9-hydroxy-10,12,15-octadecatrienoic acid. Mass fragments in black from experimental and in green from GNPS database.
